# Opsin gene evolution in aquatic, amphibious and terrestrial blennies (Blenniidae)

**DOI:** 10.1242/bio.062593

**Published:** 2026-07-15

**Authors:** Fabio Cortesi, Karen L. Cheney, Georgina M. Schlub, Zuzana Musilova, Terry J. Ord

**Affiliations:** ^1^ School of the Environment, The University of Queensland, Brisbane, QLD 4072, Australia; ^2^ Centre for Marine Science, The University of Queensland, Brisbane, QLD 4072, Australia; ^3^ Evolution and Ecology Research Centre and the School of Biological, Earth and Environmental Sciences, University of New South Wales, Sydney, NSW 2052, Australia; ^4^ Graduate School of Health, University of Technology, Sydney, NSW 2052, Australia; ^5^ Department of Zoology, Faculty of Science, Charles University, Vinicna 7, 12844 Prague, Czech Republic

**Keywords:** Fish, Vision, Transcriptomics, Gene duplication, Visual pigment

## Abstract

Evolutionary adaptations to life on land include changes to an animal's physiology, morphology and behaviour. The visual systems of amphibious fishes show pronounced morphological adaptations; however, whether molecular changes also occur remains largely unknown. Here, we investigated the molecular evolution of visual opsin genes in blennies (Blenniidae), with a primary focus on the amphibious and terrestrial Salariini. Using retinal transcriptomes and amino acid comparisons in nine species from fully aquatic to terrestrial, we found limited sequence changes and no correlation between habitat and cone opsin gene expression. The ‘red-sensitive’ *lws*, the ‘green-sensitive’ *rh2a*, and two ‘blue-sensitive’ *sws2aα* and *sws2aβ* paralogues were expressed in all species, with the latter two showing pronounced phylogenetic inertia. Long-wavelength-dominated vision is likely beneficial for feeding on algae and detritus, the primary food source of most study species, and may be co-adapted to perceive red-coloured displays in terrestrial blennies. Conversely, a lack of ‘ultraviolet-sensitive’ *sws1* expression coincides with ultraviolet-absorbing lenses in blennies, which likely evolved to protect the retina from the damaging effects of short-wavelength radiation, independent of habitat. Our data suggest that, at the molecular level, the visual systems that evolved in aquatic blennies have been retained in species that have progressively transitioned onto land.

## Teaser

**Summary:** Despite repeated water-to-land transitions in blennies, driving anatomical visual adaptations in these small ground-dwelling fishes, we found no differences in visual opsin expression or structure between terrestrial and aquatic species.

## INTRODUCTION

The evolution of tetrapods provides a textbook example of the transition from water to land, which occurred approximately 380 million years ago during the Devonian period (MacIver et al., 2017). Interestingly, terrestrial or semi-terrestrial (amphibious) lifestyles have evolved independently in at least 33 osteichthyan families (Ord and Cooke, 2016; Wright and Turko, 2016). Selective forces that drive fishes to leave the water may include access to food, new or better reproductive resources (Shimizu et al., 2006), escape from aquatic predation (Ord et al., 2017), or reduced competition (reviewed in Sayer, 2005; Wright and Turko, 2016). However, plastic or evolutionary adaptations in fish sensory systems, such as olfaction (Burguera et al., 2023) and the visual system (Sayer, 2005), are often necessary to cope with the extreme environmental changes between water and air.

Since most fishes lack eyelids or similar structures, many amphibious fish species have adaptations, such as (yellow-)pigmented corneas and lenses, horizontal pupils, and retractable eyes, to cope with increased light intensity on land (Sayer, 2005). The refractive indices of air and water are also drastically different, requiring modifications to the cornea, lens, or even the retina to maintain acuity when switching between environments (Sayer, 2005). An extreme example of this is the four-eyed fish, *Anableps anableps*, which has horizontally divided eyes. The top half has a flatter cornea than the bottom half, allowing for simultaneous vision above and below water (Swamynathan et al., 2003). However, while morphological adaptations have been studied in some detail, very little is known about possible molecular changes to the visual systems of amphibious and terrestrial fishes.

At the core of animal vision lie the opsin proteins that, together with a vitamin-A-derived chromophore, form a light-sensitive photopigment located in the photoreceptors of the retina. Upon excitation by light, the photopigments undergo a conformational change, activating the phototransduction cascade, which propagates the signal to the brain (Wald, 1968; reviewed in Hunt et al., 2014). Ancestral opsin gene duplications and subsequent changes to their amino acid compositions have led to five classes of vertebrate visual opsins, which can be defined by their phylogeny, photoreceptor specificity, and the different wavelengths of light to which they are maximally sensitive (λ_max_) (Hunt et al., 2014; Baden et al., 2025). RH1, the rod opsin, is expressed in rod cells and used for dim-light vision. In contrast, the four other visual opsin classes are expressed in cone photoreceptors and used for daylight and most instances of colour vision (for an example of putative colour vision using rod opsins, see Musilova et al., 2019). These are two short-wavelength-sensitive ‘ultraviolet’ (UV) and ‘blue opsins’ (SWS1 and SWS2), one medium-wavelength-sensitive ‘green opsin’ (RH2), and a long-wavelength-sensitive ‘red opsin’ (LWS) (Yokoyama, 2002). In teleost fishes and amphibians with different cone morphologies, SWS opsins are further expressed in single cones (PR3 and PR4). In contrast, RH2 and LWS opsins are found in double cones (two fused single cones; PR1 and PR2) (Hunt et al., 2014; Baden et al., 2025).

The evolution of visual opsins in teleost fishes is particularly dynamic, involving additional gene duplications, deletions, pseudogenisations and gene conversions, which have led to fishes having between one and 40 opsin genes in their genomes (Lin et al., 2021; Musilova et al., 2021; Policarpo et al., 2025). This diversity is primarily attributed to the different light environments in which fishes live. For example, light at either end of the visible spectrum is absorbed with increasing depth, and, consequently, the genomes of deeper-living species often lack their *sws1* and *lws* genes (Musilova et al., 2019; Policarpo et al., 2025). Species living in murky red-light-dominated habitats, on the other hand, often have redshifted visual systems (e.g. Hofmann et al., 2009; Escobar-Camacho et al., 2017); however, this could also be due to sexual selection (for a review on guppy *lws* evolution, see Sandkam et al., 2018). Mutations in the opsin coding sequence, differential (co-)expression of opsin genes, switches between A_1_-based (rhodopsin) and A_2_-based (porphyropsin) chromophores, and screening pigments in the cornea, lens, and photoreceptors are additional tuning mechanisms available to fishes when adapting to changes in their visual environment (reviewed in Carleton et al., 2020).

The current study focused on the evolution of opsin genes in amphibious and terrestrial Salariini blennies, a tribe of the combtooth blennies (Blenniidae). Combtooth blennies are small (<10 cm) scaleless fish that are commonly found in many shallow tropical and warm-water marine habitats, including coral reefs, estuaries, mangroves, and tide pools, and sometimes on land (Hundt et al., 2014a; Hundt and Simons, 2018). They comprise one of the most diverse percomorph families with 400 described species (58 genera; fishbase.org) that fall into 13 phylogenetic clades (Hundt et al., 2014a; Hundt and Simons, 2018). Within the Salariini tribe, amphibious behaviour is common, and more than 20 species across at least three genera exhibit a highly terrestrial lifestyle (Ord and Cooke, 2016). In these species, post-settlement larvae (∼30 days from hatching; Platt and Ord, 2015) are believed to transition to a terrestrial lifestyle within the supralittoral splash zone and do not voluntarily return to the aquatic environment (Ord et al., 2017). Terrestrial blennies move freely over rocks, using a tail-twisting behaviour that allows them to shuffle efficiently and jump distances of several body lengths (Hsieh, 2010). Like most intertidal blennies, amphibious and terrestrial species primarily feed on detritus and algae (Hundt et al., 2014b; Hundt and Simons, 2018; Ord and Hundt, 2020) and possess cryptic body colouration and patterning that reduces predation (Morgans and Ord, 2013; Ord et al., 2017; Yao and Ord, 2025). However, unlike aquatic and most amphibious blennies, adults of terrestrial species, in both sexes, display a brightly coloured, orange-red dorsal fin during aggressive disputes and courtship (Bhikajee and Green, 2002; Shimizu et al., 2006; Ord and Hsieh, 2011). These conspicuous fins contrast against the rocky backgrounds on which the blennies are active (Morgans and Ord, 2013; Morgans et al., 2014) and are further accentuated in courting males by a darkening of the body to a largely uniform charcoal black (Ord and Hsieh, 2011).

Morphological adaptation for aerial vision amongst the Salariini has been reported for the amphibious (possibly exclusively terrestrial) Kirk's blenny, *Alticus kirkii*, which, by separating the cornea conjunctiva and cornea propria, has formed an additional eye chamber that is adjustable to accommodate changes in refracting indices of media (Zander, 1974). Also, the retina of the mildly amphibious rippled rockskipper, *Istiblennius edentulus* (see Ord and Cooke, 2016), shows prominent swellings and folds and a central depression into which the lens can be retracted. This allows light to focus on the back of the eye in water and air (Zander, 1974). Several subtidal and intertidal blennies, including a member of the Salariini, the jewelled blenny, *Salarias fasciatus*, also possess pigmented lenses, presumably to protect the retina from the damage caused by high-intensity UV radiation in their shallow marine environments (White et al., 2004; Siebeck and Marshall, 2007).

Nothing is known about the spectral sensitivities of Salariini photoreceptors (for a review on fish spectral sensitivities, see Schweikert et al., 2018). However, microspectrophotometry (MSP) in three blennies belonging to the subtidal Nemophini and the intertidal Almadablennius clades (Hundt et al., 2014a) revealed consistently long-wavelength-shifted visual systems (Loew and Lythgoe, 1978; White et al., 2004; Cheney et al., 2009). Moreover, in the case of the peacock blenny, *Salaria pavo*, a shift to longer-wavelength-sensitive rod photoreceptors was achieved through a mixture of A_1_- and A_2_-based chromophores (White et al., 2004). CYP27C1 has been identified as the enzyme that converts A_1_ to the longer-shifted A_2_-chromophore in zebrafish (Enright et al., 2015). Gene expression of *cyp27c1* has also been linked to longer-shifted visual systems in neotropical cichlids (Härer et al., 2018). Whether long-wavelength sensitivity is also common amongst Salariini species, and if this might be caused by the expression of longer-tuned opsin genes or by a chromophore shift remains to be investigated.

Regarding the molecular basis of vision, little is known about the evolution of opsin genes in blennies, and even less is known about them in Salariini species. To the best of our knowledge, our previous work on the evolution of *sws2* and *rh2* in percomorph fishes is the only one to include a Salariini species (Cortesi et al., 2015, 2021 preprint; Musilova and Cortesi, 2023). Genome mining in *Salariopsis fluviatilis* and a member of the Almadablennius clade, the rock-pool blenny, *Parablennius parvicornis*, revealed that blennies have up to six cone opsin genes (*sws1*, *sws2aα*, *sws2aβ*, *rh2a*, *rh2b*, and *lws*) and one rod opsin (*rh1*) (Cortesi et al., 2015; Musilova et al., 2019; Musilova and Cortesi, 2023). These findings were corroborated by retinal transcriptome sequencing in three aquatic species, two fangblennies belonging to the Nemophini tribe, *Plagiotremus rhinorhynchos* and *Meiacanthus atrodorsalis*, and the bicolor blenny, *Ecsenius bicolor* (Musilova et al., 2019; Stieb et al., 2023). Given that amphibious species show morphological adaptations for vision in air, blenny visual opsins are diverse, and their visual systems seem to be long-wavelength-shifted generally, we hypothesised that exposure to full sunlight (in shallow aquatic, intertidal and supralittoral habitats) caused adaptations in gene expression or amino acids in the Salariini opsin gene repertoires. More specifically, because high UV radiation causes tissue damage to the eyes (Zagarese and Williamson, 2001; Ivanov et al., 2018), we expected to observe reduced *sws1* expression in the eyes of these fish. Instead, feeding on detritus and algae (Hundt et al., 2014b), which generally show strong reflection in the long wavelength (red spectrum) due to their chlorophyll component (Stieb et al., 2017), as well as the use of red dorsal fins for aggressive and courtship displays in terrestrial species (Ord and Hsieh, 2011; Morgans and Ord, 2013), might have caused these blennies to have a more long-wavelength-dominated, *lws*, visual system.

To test whether Salariini opsins showed adaptation to living outside the water, we collected and sequenced the retinal transcriptomes of six Salariini and three additional blenny species. These represented three of the four major blenny clades and ranged in habitats from fully aquatic (i.e. subtidal) to amphibious and terrestrial species (i.e. intertidal). Analysing opsin gene expression within a phylogenetic comparative framework, we found no evidence that habitat drives gene expression. Likewise, opsin-gene-coding regions showed little variation between species, and no sites were found to be under positive selection. However, we did discover a strong correlation between opsin gene expression and phylogeny.

## RESULTS

### Opsin gene phylogeny and gene expression

**Fig. 1.**
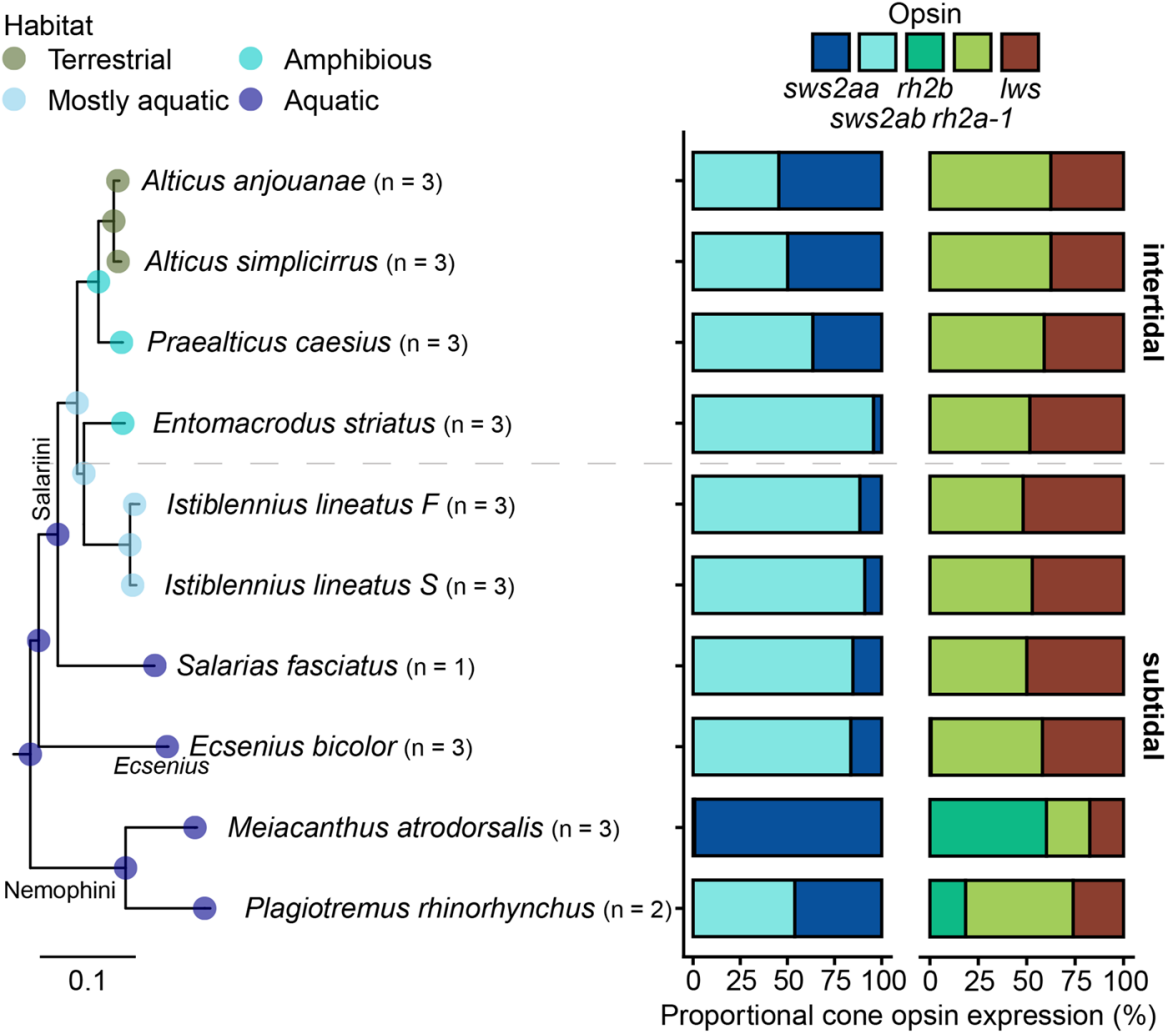
**Cone opsin gene expression in blennies.** The mean proportional cone opsin gene expression separated by single [short-wavelength-sensitive 2 (*sws2*)] and double [mid-wavelength-sensitive (*rh2*) and long-wavelength-sensitive (*lws*)] cones is mapped onto the newly generated transcriptome-based blenny phylogeny. Intertidal and subtidal blennies from this study fall into three major Blenniidae tribes: Nemophini, *Ecsenius*, and Salariini (Hundt et al., 2014a). Habitat type and ancestral states are as per Ord and Cooke (2016). Note that, except for the Nemophini, blennies show highly similar, long-wavelength-dominated expression profiles (also see Table 1). *Istiblennius lineatus* S=Seychelles, *I. lineatus* F=French Polynesia.

**Fig. 2.**
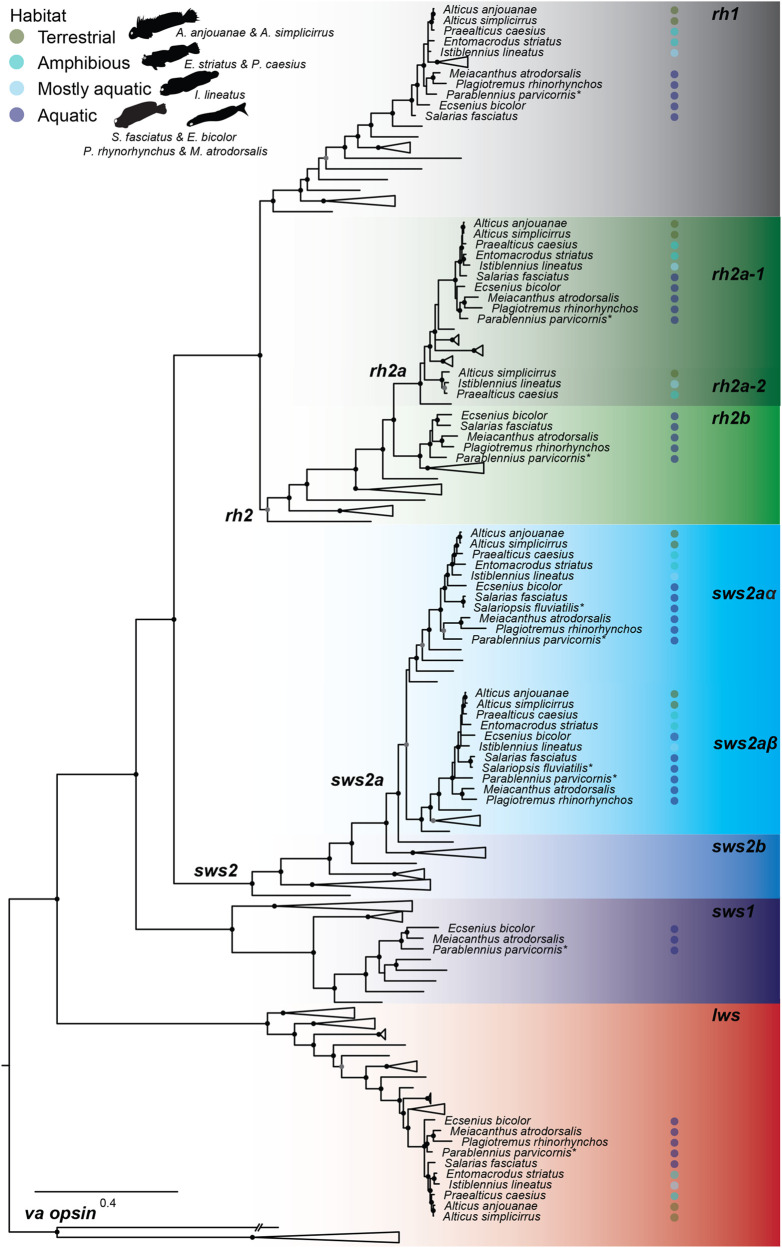
**Blenniidae visual opsin gene phylogeny.** The blenny retinal transcriptomes contained eight visual opsin genes. One rod opsin (*rh1*) and seven cone opsin genes belonging to four different cone opsin classes. The single-cone opsins, short-wavelength-sensitive 1 (*sws1*) and 2 (*sws2aα*, *sws2aβ*), and the double-cone opsins, rhodopsin-like 2 (*rh2a-1*, *rh2a-2*, *rh2b*) and long-wavelength-sensitive (*lws*). Note that *rh2a-2* was found at trace expression levels in only three out of the six Salariini species, while *sws1* was expressed at <0.5% of total cone opsin gene expression in *Ecsenius bicolor* and *Meiacanthus atrodorsalis* (also see Table 1). Black and grey spheres indicate Bayesian posterior probabilities of >0.9 and 0.7, respectively. *Salariopsis fluviatilis and *Parablennius parvicornis* genes from Cortesi et al. (2015) and Musilova et al. (2019), respectively. A detailed phylogeny and GenBank accession numbers are shown in Fig. S1 and Table S1.

Retinal transcriptomes from the species we studied contained seven cone opsins and one rod opsin (*rh1*) (Fig. 1). Phylogenetic reconstruction revealed one UV-sensitive *sws1* gene, two blue-sensitive *sws2a* copies (*sws2aα* and *sws2aβ*), three green-sensitive *rh2* copies (*rh2a-1*, *rh2a-2*, and *rh2b*), and one red-sensitive *lws* opsin gene (Fig. 2; Fig. S1, Table S1).

**Table 1.** Summary of transcriptomes, opsin mapping, and proportional opsin gene expression

RNA sequencing					Proportional opsin and *cyp27c1* expression (%)										
Transcriptome			Rod versus cone		Cone opsin versus total cone expression					Single cones		Double cones			*cyp27c1*
Species	ID	Number of raw reads	*rh1*	Total cones	*sws2a*		*rh2b*	*rh2a-1*	*lws*	*sws2a*		*rh2b*	*rh2a-1*	*lws*	
					*α*	*β*				*α*	*β*				
*Alticus simplicirrus* French Polynesia Terrestrial	B7	20,291,698	85.7	14.3	7.2	6.5	−	60.1	26.2	52.2	47.8	−	69.7	30.3	0.15
	B8	15,763,272	84.9	15.1	5.6	5.3	−	53.0	36.2	51.4	48.6	−	59.4	40.6	0.13
	B9	22,851,082	83.1	16.9	6.2	4.1	−	52.1	37.6	60.2	39.8	−	58.1	41.9	0.14
	Mean	19,635,351	84.6	15.4	6.3	5.3	−	55.1	33.3	54.6	45.4	−	62.4	37.6	0.14
	s.e.	2,072,226	0.8		0.5	0.7	−	2.5	3.6	2.8		3.7			0.01
*Alticus anjouanae* Seychelles Terrestrial	B10	22,500,526	92.4	7.6	7.7	6.0	−	57.8	28.6	56.3	43.7	−	66.9	33.1	0.06
	B11	37,810,316	91.8	8.2	5.9	6.1	−	52.8	35.2	49.3	50.7	−	60.0	40.0	0.11
	B12	6,118,452	90.7	9.3	5.9	7.5	−	52.5	34.1	44.1	55.9	−	60.6	39.4	0.18
	Mean	22,143,098	91.6	8.4	6.5	6.5	−	54.3	32.6	49.9	50.1	−	62.5	37.5	0.12
	s.e.	9,150,398	0.5		0.6	0.5	−	1.7	2.0	3.5		2.2			0.03
*Praealticus caesius* French Polynesia (Mildly amphibious)	B4	19,353,526	94.5	5.5	9.8	14.6	−	55.7	19.9	40.2	59.8	−	73.7	26.3	0.12
	B5	19,211,400	89.7	10.3	5.0	8.3	−	47.3	39.5	37.4	62.6	−	54.5	45.5	0.15
	B6	27,900,670	74.3	25.7	4.8	10.2	−	41.5	43.6	32.1	67.9	−	48.8	51.2	0.03
	Mean	22,155,199	86.1	13.9	6.5	11.0	−	48.2	34.3	36.6	63.4	−	59.0	41.0	0.10
	s.e.	2,873,029	6.1		1.6	1.9	−	4.1	7.3	2.4		7.5			0.04
*Entomacrodus striatus* Seychelles Amphibious	B13	27,001,052	49.6	50.4	1.9	9.5	−	42.0	46.6	16.4	83.6	−	47.5	52.5	0.02
	B14	30,325,336	50.6	49.4	1.1	10.4	−	41.1	47.4	9.7	90.3	−	46.5	53.5	0.01
	B15	20,757,818	46.5	53.5	1.0	11.2	−	44.1	43.6	8.3	91.7	−	50.3	49.7	0.02
	Mean	26,028,069	48.9	51.1	1.3	10.4	−	42.4	45.8	11.5	88.5	−	48.1	51.9	0.02
	s.e.	2,804,423	1.2		0.3	0.5	−	0.9	1.1	2.5		1.1			0.00
*Istiblennius lineatus* French Polynesia Aquatic (Mildly amphibious)	B1	11,510,186	77.0	23.0	1.1	14.3	−	50.0	34.6	7.0	93.0	−	59.1	52.5	0.02
	B2	6,765,526	71.6	28.4	1.2	12.3	−	49.9	36.6	9.1	90.9	−	57.7	53.5	0.01
	B3	3,343,260	73.0	27.0	1.5	12.7	−	49.4	36.4	10.5	89.5	−	57.6	49.7	0.02
	Mean	7,206,324	73.9	26.1	1.3	13.1	−	49.8	35.9	8.9	91.1	−	58.1	51.9	0.02
	s.e.	2,367,868	1.6		0.1	0.6	−	0.2	0.6	1.0		0.5			0.11
*Istiblennius lineatus* Seychelles Aquatic (Mildly amphibious)	B16	30,354,518	58.1	41.9	0.6	13.0	−	48.2	38.2	4.6	95.4	−	55.8	52.5	0.02
	B17	56,930,548	57.0	43.0	0.6	13.5	−	47.1	38.8	4.2	95.8	−	54.8	53.5	0.01
	B18	7,165,396	55.3	44.7	0.6	13.5	−	47.3	38.6	4.4	95.6	−	55.1	49.7	0.02
	Mean	31,483,487	56.8	43.2	0.6	13.3	−	47.5	38.5	4.4	95.6	−	55.2	51.9	0.02
	s.e.	14,377,048	0.8		0.0	0.2	−	0.3	0.2	0.1		0.3			0.01
*Salarias fasciatus* Australia Aquatic	SF1 HRS 2017	44,650,228	79.9	20.1	2.3	13.0	−	42.3	42.4	15.3	84.7	−	50.0	50.0	0.09
*Ecsenius bicolor* Australia Aquatic	FB10	25,890,490	45.6	54.4	1.5	13.8	0.5	41.9	42.4	9.6	90.4	0.6	49.4	50.0	0.01
	FB2	43,286,456	54.8	45.2	2.3	12.0	0.5	46.4	38.8	16.1	83.8	0.6	54.1	45.3	0.00
	C4	8,771,964	88.2	11.8	4.7	15.1	0.4	55.4	24.5	23.6	76.4	0.5	69.0	30.5	0.06
	Mean	25,982,970	62.9	37.1	2.8	13.6	0.5	47.9	35.2	16.4	83.5	0.6	57.5	41.9	0.02
	s.e.	8,135,231	13.0		1.0	0.9	0.0	4.0	5.5	4.0		0.0	5.9	5.9	0.02
*Meiacanthus atrodorsalis* Australia Aquatic	FD9	12,751,878	31.5	68.5	14.5	0.0	42.8	21.3	20.9	97.3	0.1	50.4	25.1	24.6	0.0
	GA1	65,018,820	45.9	54.1	15.1	0.0	50.1	20.3	14.5	99.7	0.2	59.0	23.9	17.1	0.0
	S209	64,527,374	62.6	37.4	16.4	0.3	59.3	15.1	8.9	97.9	2.0	71.2	18.1	10.7	0.0
	Mean	47,432,691	46.7	53.3	15.3	0.1	50.7	18.9	14.8	98.3	0.8	60.2	22.4	17.4	0.0
	s.e.	14,158,856	9.0		0.6	0.1	4.8	1.9	3.5	0.7	0.6	6.1	2.2	4.0	0.0
*Plagiotremus rhinorhynchos* Australia Aquatic	FZ7	19,391,482	46.3	53.7	12.5	12.9	13.8	49.6	11.3	49.2	50.8	18.5	66.4	15.1	0.0
	FZ8	15,688,728	28.0	72.0	7.6	10.0	15.1	36.8	30.5	43.0	57.0	18.3	44.7	37	0.0
	Mean	17,540,105	37.2	62.8	10.0	11.5	14.4	43.2	20.9	46.1	53.9	18.4	55.5	26.1	0.0
	s.e.	1,309,121	8.3		2.3	1.1	1.2	5.1	7.5	3.1		0.1	10.8	11	0.0

Number of raw reads refers to the total number of paired-end fragments. *rh1*=rod opsin, *sws2*=short-wavelength sensitive 2, *rh2*=rhodopsin like 2, *lws*=long-wavelength sensitive. Single cones refer to PR3 and PR4, and double cones refer to PR1 and PR2, as per Baden et al. (2025), with double cones being two fused single cones.

Rod opsin expression in Salariini species and *E. bicolor* was higher than cone opsin expression (>60% of total opsin expression), except in *Entomacrodus striatus* and *Istiblennius lineatus* (S), in which, like the two fangblennies *M. atrodorsalis* and *Pl. rhinorhynchos*, rod and cone opsin genes were more equally expressed (∼50% of total opsin expression each) (Fig. 1, Table 1).

In terms of single-cone opsin expression (PR3 and PR4 *sensu*; Baden et al., 2025), we found traces of *sws1* expression (<0.5% of total cone opsin expression) in *M. atrodorsalis* and *E. bicolor* (Fig. 2). These low levels of expression are likely derived from the relatively few larval cones that remain at the centre of the retina as the eye grows in development and switches to the adult opsin repertoire (Musilova et al., 2021). Hence, in the adults analysed here, most single-cone gene expression was *sws2a* based, accounting for ∼11–20% of total cone opsin expression (Fig. 1, Table 1). In single cones, the *sws2a* paralogues were expressed at similar ratios (∼40–60% each) within the subclade containing the two terrestrial *Alticus* spp. and the mildly amphibious *Praealticus caesius* and in the aquatic *Pl. rhinorhynchos*. *M. atrodorsalis* almost exclusively expressed *sws2aα*, while in the remaining species, *sws2aβ* was primarily expressed (>83%) (Fig. 1, Table 1). The violet-sensitive *sws2b* gene was not expressed in the retinae of our study species (Fig. 2; Fig. S1).

Considering double-cone gene expression (PR1 and PR2 *sensu*; Baden et al., 2025), all Salariini blennies and *E. bicolor* were found to express high levels of *rh2a-1* and *lws* (∼30–60% each) (Fig. 1, Table 1). *Rh2a-2*, on the other hand, was found to be lowly expressed (<0.1%), only allowing full-coding-sequence reconstruction for *Alticus simplicirrus* and partial sequence reconstructions for *Pr. caesius* and *I. lineatus* (Fig. 2; Fig. S1). *Rh2b* was found to be expressed in trace amounts in *S. fasciatus* and in the non-Salariini blenny *E. bicolor*, ∼14% in *Pl. rhinorhynchos* and ∼50% in *M. atrodorsalis* (Fig. 1, Table 1).

The gene encoding the enzyme that converts A_1_ to the A_2_ chromophore, *cyp27c1* (Enright et al., 2015), was found to be weakly expressed in all Salariini species and in *E. bicolor*, at <0.5% of total opsin expression. It was not expressed in the two fangblenny species (Table 1).

### Blenny phylogeny and phylogenetic comparative analyses

A high level of support (Bayesian posterior probability of 1) was found for all internal nodes and the branches leading to extant species in the transcript-based blenny phylogeny (Fig. 1). The phylogenetic relationships among our target species were largely congruent with those of Hundt et al. (2014a). Within the Salariini, *Alticus* and *Praealticus* formed a sister clade to the *Istiblennius*-*Entomacrodus* clade; the two *Istiblennius* populations (French Polynesia and Seychelles) also formed a sister clade. The main difference was with the placement of *Ecsenius* and the Nemophini (fangblennies). Our reconstruction placed *Ecsenius* as the sister of the Salariini, with Nemophini basal within the Blenniidae, whereas in Hundt et al. (2014a), their position could not be resolved with certainty.

Independent of whether habitat was analysed as aquatic versus amphibious versus terrestrial or as tidal versus subtidal, there was no significant relationship between habitat and opsin gene expression in blennies, as assessed by the phylogenetic generalised least squares (PGLS) regression approach. However, opsin gene expression showed a strong phylogenetic dependence for the two *sws2a* paralogues, with Pagel's λ ranging from 0.98 to 1, and for *lws*, with Pagel's λ=1 for all comparisons. No phylogenetic dependence was discovered for the *rh2a-1* gene (Table S2).

### Opsin gene sequence analyses

The Salariini opsin coding sequences were highly similar when compared between species within the clade (SWS2Aα=95–100% similarity, SWS2Aβ=96–100%, RH2A-1=98–100%, LWS=98–100%, RH1=97–100%), showed a slightly increased variability when compared to the *E. bicolor* orthologues (SWS2Aα=94–95%, SWS2Aβ=97–99%, RH2A-1=97–98%, LWS=96–97%, RH1=96–97%), and had the highest variability with the orthologues of the two Nemophini, fangblenny species (SWS2Aα=89–92%, SWS2Aβ=94–96%, RH2A-1=94–95%, LWS=97–98%, RH1=95–97%) (Table S4).

When comparing substitutions within transmembrane sites, the patterns were not as clear (Table S4). The Nemophini species showed the highest number of substitutions compared to the consensus sequence for SWS2Aα (*n*=25 and *n*=15 for the Salariini and *n*=8 for *E. bicolor*) and SWS2Aβ (*n*=23, 10, and 4), followed by RH2A-1 (*n*=18, 3, and 5). LWS (*n*=4, 10, and 3) showed an increased number of substitutions in the Salariini, while RH1 (*n*=9, 11, and 7) had a similar number of substitutions between all three tribes.

When examining the retinal binding pocket alone, there were few changes compared to the overall blenny consensus sequence (Table S4). SWS2Aα showed the highest number with two substitutions that changed the polarity of the amino acid in Salariini and *E. bicolor* versus the more basal Nemophini (S68A, T299A; amino acid numbering as per the reference bovine RH1), plus non-polarity-affecting changes in *Pl. rhinorhynchos* C95F, L273V in *M. atrodorsalis*, and L262I in the Salariini *S. fasciatus*. SWS2Aβ had one non-polarity-changing substitution, T52S, in the Salariini and *E. bicolor*, except for *I. lineatus*, which, like the Nemophini, had a threonine at that site. For RH2A, *Pl. rhinorhynchos* had two polarity-affecting amino acid changes in A98S and A124S, and *M. atrodorsalis* and *E. bicolor* also showed a minor substitution in I179L. Only one amino acid substitution was found to occur in the retinal binding pocket of LWS, with S164A being a polarity-changing substitution in *Pr. caesius*. Finally, for RH1, a major substitution affecting both the polarity and charge of the site was found in N83D in the Salariini, with minor changes in A124G in Salariini *S. fasciatus* and V189I in *E. bicolor* and Salariini *E. striatus*.

Only a few amino acid substitutions between species were found to occur in potential spectral tuning sites, namely those previously identified to change the spectral properties of opsin pigments and/or substitutions that incorporated changes in polarity (reviewed in Hagen et al., 2023; Table S4). Of particular interest in SWS2Aα was site I46F, from the basal *Pl. rhinorhynchos* to the more derived blennies, with a similar substitution in killifish (L46F) resulting in a −6 nm blueshift (Yokoyama et al., 2007). Also, SWS2Aα G109A and T116M in the Salariini and *E. bicolor*, when mutating in the reverse direction, from A109G and I116T in killifish, cause a −2 and −7 nm shift, respectively (Yokoyama et al., 2007). In LWS, site S164A in *Pr. caesius* stood out as it is known to blueshift the peak spectral sensitivity of the visual pigment by −7 nm (Yokoyama, 2008). For RH1, site N83D in Salariini versus Nemophini and *E. bicolor* has been found to redshift the visual pigment by +2 nm (Yokoyama, 2008), and site A124G in *S. fasciatus* is known to redshift the visual pigment by +1–3 nm (Hunt et al., 2001). Overall, the Salariini visual opsins show low variability. Salariini genes show low variability. Reflecting this, analyses using the newly generated blenny phylogeny (Fig. 1) to run CODEML in PAML (Yang, 2007) for the various opsin genes revealed that none of the Salariini genes is currently evolving under positive selection (Table S3).

## DISCUSSION

In this study, we investigated the molecular evolution of vision in blennies, with a particular focus on the Salariini tribe, which comprises multiple species that have transitioned from aquatic to terrestrial environments. First, we created a new species phylogeny based on the retinal transcriptomes of nine blenny species classified as aquatic (Nemophini: *Pl. rhinorhynchos*, *M. atrodorsalis*; Salariini: *S. fasciatus*; and *E. bicolor*), mostly aquatic (Salariini: *I. lineatus* and *Pr. caesius*), amphibious (Salariini: *Pr. caesius*, *E. striatus*), and terrestrial (Salariini: *A. simplicirrus* and *Alticus anjouanae*) (Ord and Cooke, 2016; Fig. 1). Using opsin gene expression, amino acid predictions, phylogenetic modelling and a phylogenetic least squares analysis, we found that, despite various degrees of phylogenetic inertia (Table S2), there was little variability in visual opsin sequences (Tables S3 and S4) and gene expression (Fig. 1, Table 1) between species; blennies expressed one rod opsin (*rh1*) and between three and four cone opsins (Fig. 2), independent of habitat or geographic region (i.e. South Pacific versus Indian Ocean).

*Rh1* expression in fishes shows a strong diurnal pattern, starting with a high expression in the morning and gradually decreasing throughout the day (Korenbrot and Fernald, 1989; Stieb et al., 2016; Yourick et al., 2019). Hence, differences in sampling time between early morning and late afternoon most likely explain the discrepancy in rod-to-cone opsin expression observed in this study (Table 1).

Regarding the cone opsins, all Salariini species expressed the longer-shifted double- (*lws* and *rh2a-1*) and single-cone opsins (*sws2a*s). We also discovered a second *rh2a* paralogue in the genera *Alticus*, *Praealticus*, and *Istiblennius* (Fig. 1)*.* The *rh2a-2* gene was not expressed in the closely related *S. fasciatus* or in the more distantly related Nemophini and *E. bicolor* (Musilova et al., 2019). Since it is also absent from the genome of *Pa. parvicornis* (Musilova et al., 2019), one could assume that this *rh2a* duplication is specific to the clade containing the amphibious and terrestrial Salariini species (Ord and Cooke, 2016). However, based on our phylogenetic reconstruction, *rh2a-2* is basal to a greater *rh2a* clade, which includes gene orthologues from dottybacks and cichlids (Fig. S1). Both cichlids (Escobar-Camacho et al., 2017) and dottybacks (Cortesi et al., 2016) have two *rh2a* duplicates, which have undergone widespread gene conversion and, consequently, cluster closely together within their respective species/families. The blenny *rh2a* copies, on the other hand, appear unaffected by gene conversion. Rather than being Salariini specific, then, it is likely that *rh2a-2* is the blenny orthologue of an ancestral *rh2a* duplication and that *Pa. parvicornis* (and possibly also the species lacking expression) has lost the copy secondarily. As more and more fish genomes become available (e.g. Musilova et al., 2019; Policarpo et al., 2025), the patterns surrounding *rh2* evolution are likely to become clearer in the near future (also see Musilova and Cortesi, 2023, for a recent attempt to resolve *rh2* evolution in teleosts).

Finally, a strong phylogenetic signal was observed in the expression of the short-wavelength-sensitive *sws2a* duplicates (Fig. 1, Table 1; Table S2). The clade containing the two terrestrial *Alticus* species and the amphibious *Pr. caesius* expressed both copies at a similar ratio, as did the aquatic *Pl. rhinorhynchos* (Fig. 1, Table 1). The remaining Salariini species and *E. bicolor* primarily expressed the *sws2aβ* paralogue, whereas *M. atrodorsalis* predominantly expressed *sws2aα* (Fig. 1, Table 1). In dottybacks and other percomorphs, *SWS2Aα* is ∼10 nm shorter shifted compared to *SWS2Aβ* (Cortesi et al., 2015). Adult dottybacks express both copies, forming two distinct photoreceptors that are thought to increase their ability to discriminate between the differently coloured fish they mimic (Cortesi et al., 2016). Alternatively, some fishes have been found to co-express two single-cone opsins within the same photoreceptor, with opposite implications depending on the species in question; in cichlids, single-cone co-expression is thought to increase achromatic (brightness) contrast at the cost of chromatic (colour) contrast (Dalton et al., 2017), while in anemonefishes, co-expression seems to increase chromatic contrast (Stieb et al., 2019). Fluorescence *in situ* hybridisation, MSP, and, ultimately, behavioural experiments will be needed to test whether the difference in *sws2a* expression observed here is ecologically significant or the result of phylogenetic inertia.

Opsin gene expression in fishes has been shown to be influenced by several factors, including changes in the light environment on both macrohabitat (e.g. Fuller et al., 2005; Stieb et al., 2016) and microhabitat scales (e.g. Luehrmann et al., 2020) (reviewed in Shaughnessy and Cortesi, 2025). However, our data show no major differences in opsin gene expression among blenny species from different habitats (Fig. 1, Table 1). Moreover, opsin orthologues were highly conserved between blenny species, and only very few changes in retinal binding pocket sites and/or key tuning sites, potentially shifting spectral sensitivities by small degrees (from −7 nm in SWS2Aα and LWS to +3 nm in RH1), could be found (Table S4). Although the amino acid variability was somewhat higher between the Salariini tribe and other blenny species, opsin gene expression in all species showed a highly congruent, long-wavelength-shifted profile (also see Musilova et al., 2019; Musilova and Cortesi, 2023). Hence, neither at the expression nor at the opsin-sequence level were there any apparent adaptations to the transition from water to land, at least for the limited species we had access to. This may not be surprising, since the blenny species analysed here all experience a broad, high-intensity light spectrum with only, if any, subtle differences in the light environment between shallow-reef and supralittoral habitats. However, the findings in the blennies contrast with those in mudskippers (Gobiidae), which have convergently evolved an amphibious lifestyle, in which adaptations in opsin gene expression and increases in structural variation have been reported (You et al., 2014; Cortesi et al., 2021 preprint). Mudskippers do not express the green-sensitive RH2 opsin; instead, the functional niche is filled by a second, green-sensitive LWS paralogue. This LWS paralogue (LWS-3) carries more amino acid mutations, resulting in a stronger green shift of the visual pigment compared to its orthologue in the aquatic gobies (Cortesi et al., 2021 preprint). This shows that different molecular adaptations can drive the convergent evolution of amphibious and terrestrial lifestyles between fish families.

Interestingly, *cyp27c1*, the gene that encodes the enzyme that converts A_1_ (rhodopsin) to the A_2_ (porphyropsin) chromophore (Enright et al., 2015), was lowly expressed in all species except the Nemophini fangblennies (Table 1). This might be due to phylogeny, or, alternatively, the use of porphyropsin may have served as an early adaptation to the broader light environment of shallow and intertidal marine waters, especially as the fangblennies analysed here occur in slightly deeper, bluer reef environments. Moreover, as shown in the peacock blenny, a species that lives along the tidal line, the longer-shifted porphyropsin was used differently between the sexes (White et al., 2004). Hence, differences in sensitivity to longer wavelengths of light might have facilitated signalling in species with elaborate, often red or orange, sexually dimorphic ornaments, such as the peacock blenny and members of the Salariini tribe (Ord and Hsieh, 2011).

Mounting evidence also suggests that long-wavelength sensitivity in coral reef fishes is especially beneficial when feeding on algae or similar chlorophyll-containing organic matter, which strongly reflects long-wavelength light (reviewed in Marshall et al., 2018). It is also possible that the red-orange dorsal fin that appears unique to the terrestrial species (Yao and Ord, 2025) has evolved to exploit the pre-existing visual state of seeing red as a means of maximising the efficiency of territorial and courtship signalling on land (Bhikajee and Green, 2002; Shimizu et al., 2006; Ord and Hsieh, 2011). The red-orange colouration of the dorsal fin of terrestrial blennies has been shown to be highly chromatically contrasting with their typical background (Morgans and Ord, 2013; Yao and Ord, 2025). Similarly, the evolution of sexually selected red-orange colour signals in response to an innate red sensory bias in conspecific receivers has also been suggested for a number of freshwater fish species [e.g., guppies (Rodd et al., 2002), sticklebacks (Smith et al., 2004), and cichlids (Seehausen et al., 2008)]. Likewise, in the marine realm, many damselfishes and other reef fishes that feed on algae also use red for signalling (Stieb et al., 2023).

It is notable that none of the species expressed the violet-sensitive *sws2b* gene, and only *E. bicolor* and *M. atrodorsalis* expressed the UV-sensitive *sws1* at very low levels (Fig. 2, Table 1). It is possible that *sws2b* was lost in the ancestor of all blennies (Cortesi et al., 2015). Salariini blennies could have lost *sws1* and *rh2b* (also not expressed in Salariini; Fig. 1, Table 1) independently, or alternatively, these genes might simply not be expressed in adult blennies. Instead, the genes (and *rh2a-2*) may be utilised at different developmental stages, a common feature of visual opsin gene expression in fishes (e.g. Spady et al., 2006; Carleton et al., 2008; Cortesi et al., 2016; Savelli et al., 2018). Supporting the potential use of *sws1* at earlier life stages, Siebeck and Marshall (2007) found that adult Salariini blennies have UV-blocking eyes, whereas larval stages have eyes that transmit UV to violet light. In an interesting parallel, mudskippers (You et al., 2014) and the Asian swamp eel (*Monopteros albus*) (Musilova et al., 2019), both of which are amphibious, have lost their *sws1* gene, with the swamp eel further missing its *sws2b* copy (Cortesi et al., 2015). It is possible that the tissue damage induced by UV radiation (Zagarese and Williamson, 2001; Ivanov et al., 2018) has led to the convergent loss and/or inactivation of the shorter-shifted *sws* genes in shallow-water and amphibious species more generally.

In summary, we found no apparent molecular adaptations in visual opsin gene sequence variability and expression in terrestrial Salariini species compared to their amphibious and aquatic sister species or to more distantly related species. On the contrary, our data suggest that (Salariini) blennies evolved visual systems early on that were ideal to conquer shallow reef and intertidal habitats, and very few, if any, molecular adaptations have been made for life on land.

## MATERIALS AND METHODS

### Study species

Adult blennies belonging to the Salariini clade (Hundt et al., 2014a) were collected on snorkel or on foot with hand nets from several sites around the Indo-Pacific. Given that these blennies actively seek shade to avoid desiccation (Ord and Hsieh, 2011), sampling occurred either early in the morning or late in the afternoon. Immediately post-capture, their eyes were enucleated and stored in RNAlater (Life Technologies) for subsequent molecular analysis. From French Polynesia, we collected lined rockskipper, *I. lineatus* (Valenciennes, 1836) (Mo'orea: 17°29′52.05″S, 149°45′17.24″W; 8–10 November 2013; *N*=3); blackmargin rockskipper, *Pr. caesius* (Seale, 1906) (Mo'orea: 17°29′52.05″S, 149°45′17.24″W; 8–10 November 2013; *N*=3); and Marquesan rockskipper, *A. simplicirrus* (Smith-Vaniz and Springer, 1971) (Tahiti: 17°30′57.67″S, 149°24′25.14″W; 14–15 November 2013; *N*=3). From the Seychelles, we collected *I. lineatus* (4°48′9″S, 53°30′60″E and 4°33′56″S, 55°27′10″E; 19 and 26 April 2014; *N*=3); reef margin blenny, *E. striatus* (Valenciennes, 1836) (4°48′9″S, 53°30′60″E; 19 and 22 April 2014; *N*=3); and the Seychelles rockskipper, *A. anjouanae* (Fourmanoir, 1955) (4°33′56″S, 55°27′10″E; 20 April 2014; *N*=3). A jewelled blenny, *S. fasciatus* (Bloch, 1786) (*N*=1), was collected in May 2017 from Heron Island (23°44′S, 151°91′E), Southern Great Barrier Reef, Australia.

The dataset was complemented with retinal transcriptomes from three non-Salariini blenny species that were sequenced as part of our previous work on vision in coral reef fishes: bicolor blenny, *E. bicolor* (Day, 1888) (*N*=3); forktail blenny, *M. atrodorsalis* (Günther, 1877) (*N*=3) (both Stieb et al., 2023); and the bluestriped fangblenny, *Pl. rhinorhynchos* (Bleeker, 1852) (*N*=2) (Musilova et al., 2019). These fishes were collected from reefs around Lizard Island, Northern Great Barrier Reef, Australia (14°40′08″S, 145°27′34″E) (Fig. 1).

All experimental procedures were approved by the Animal Ethics Committees from the University of New South Wales (11/36B and 13/21B) and The University of Queensland (QBI/304/16). Fish were collected under permits issued by Protocole D'Accueil (10 October 2013; French Polynesia), the Seychelles Bureau of Standards (#A0157), the Great Barrier Reef Marine Park Authority (G17/38160.1), and Queensland Fisheries (#180731).

### Transcriptome sequencing, quality filtering, and *de novo* assembly

Retinae were dissected from the eyecups, and total RNA was extracted using an RNeasy Mini Kit (Qiagen), including DNase treatment, according to the manufacturer's instructions. RNA integrity was checked using a Eukaryotic Total RNA Nano chip on a Bioanalyzer 2100 (Agilent Technologies). Newly acquired retinal transcriptomes, except for *S. fasciatus*, were sequenced in-house at the Queensland Brain Institute's sequencing facility, Brisbane, Australia. Sequencing libraries were prepared from 100–1000 ng of total RNA using the TruSeq total-stranded mRNA Library Prep Kit protocol (Illumina, San Diego), and library concentrations were measured using a Qubit dsDNA BR Assay Kit (Thermo Fisher Scientific). Individual libraries were barcoded, and up to 12 libraries per lane were pooled at equimolar ratios. Libraries were sequenced at paired-end PE125 on a HiSeq 2000 using Illumina's SBS chemistry version 4. The *S. fasciatus* library preparation (strand-specific, 300 bp insert) and sequencing (RNA-seq HiSeq PE150) were outsourced to Novogene (https://en.novogene.com/).

Filtering and *de novo* assembly of retinal transcriptomes followed the protocols described by de Busserolles et al. (2017) and Tettamanti et al. (2019). In short, raw-read transcriptomes were uploaded to the Genomics Virtual Laboratory (GVL 4.0.0) (Afgan et al., 2015) on the Galaxy Australia server (https://usegalaxy.org.au/) and quality filtered using Trimmomatic (Galaxy v.0.32.2) (Bolger et al., 2014) before being *de novo* assembled using Trinity (Galaxy v.0.0.2) (Haas et al., 2013). At least two transcriptomes per species were assembled, except for *S. fasciatus*, where only one individual was sequenced (Table 1).

### Opsin gene mining, gene tree reconstructions, and opsin gene expression

Opsin gene mining and expression analyses followed the protocol described by de Busserolles et al. (2017). In short, putative blenny opsin genes were searched for by mapping the assembled transcripts to the opsin coding sequences of the dusky dottyback, *Pseudochromis fuscus* (Cortesi et al., 2016) in Geneious v.11.0.2 (www.geneious.com). *Ps. fuscus* was chosen because it belongs to the closely related family Pseudochromidae (Alfaro et al., 2018) and possesses an opsin gene repertoire that includes representatives of all ancestral vertebrate opsin gene classes (Cortesi et al., 2016). Mapped contigs were extracted and compared to publicly available opsin gene sequences using BLASTN (https://blast.ncbi.nlm.nih.gov/Blast.cgi). Moreover, because *de novo* assembly based on short-read libraries is prone to creating misassemblies (chimeric sequences) and overlooking closely related or lowly expressed gene copies, we used a second approach to account for all expressed visual opsins. This approach involved mapping unassembled reads to the reference *Ps. fuscus* genes, followed by manual extraction of gene copies using paired-end information to transition from single-nucleotide polymorphisms (SNPs) to SNPs within genes. Extracted reads were *de novo* assembled, and, if necessary, their consensus was used as a species-specific template against which unassembled reads were repeatedly remapped until the entire coding region could be extracted.

Blenny visual opsins were confirmed and assigned to specific opsin gene classes based on their phylogenetic relationships to a reference dataset obtained from GenBank (https://www.ncbi.nlm.nih.gov/genbank/) and Ensembl (www.ensembl.org/), which included the blenny opsin genes mined from the *Pa. parvicornis* genome (Musilova et al., 2019) and the *sws2a* paralogues from *S. fluviatilis* (Cortesi et al., 2015) (Fig. 2; Fig. S1, Table S1). Gene coding regions were aligned using the L-INS-I settings in the Geneious MAFFT plugin v.1.3.7 (Katoh and Standley, 2013), and jModelTest v.2.1.10 (Darriba et al., 2012) was subsequently used to select the most appropriate model of sequence evolution based on the Akaike information criterion. MrBayes v.3.2.6 (Ronquist et al., 2012), run on the CIPRES platform (Miller et al., 2010), was then used to infer the phylogenetic relationship between opsin genes using the following parameter settings: GTR+I+G model; two independent Markov chain Monte Carlo searches with four chains each; 10 million generations per run; 1,000 generations sample frequency; 25% burn-in. Phylogenies were also reconstructed using GTR+G models to account for variable sites. However, no substantial differences in tree structure or node support could be found between models (UQ eSpace, https://doi.org/10.48610/09af62a).

Because visual opsin gene and *cyp27c1* expression in fishes follow a strong diurnal circadian rhythm, normalising gene expression to housekeeping genes or calculating the fold change within the transcriptome (fragments per kilobase of transcript per million mapped reads or transcripts per million) to compare between species is feasible only if sample collection is standardised to the same time of the day (Yourick et al., 2019), which was not the case in this study. Instead, as opsin expression is synchronised across the day, calculating each gene's expression as a proportion of total opsin expression or within specific cell types allows interpretation of changes in colour vision, i.e. whether an individual uses more of a specific opsin than another, which was what we were interested in. Hence, proportional opsin gene expression was measured by mapping unassembled reads against the extracted opsin coding regions for each species, as described by Tettamanti et al. (2019).

We then compared the rod opsin expression to the combined cone opsin expression and the proportional expressions of single- (*sws1*, *sws2a*'s) and double-cone (*rh2*'s, *lws*) genes amongst themselves (since the expression of those genes is constrained to different cone morphologies, PR1–PR4 as per Baden et al., 2025) (Fig. 1, Table 1). We also quantified *cyp27c1* expression by comparing it with total opsin gene expression in fishes (Table 1). This was followed by a Bayesian reconstruction using GTR+G, with or without invariable sites, as the evolutionary model to reconstruct the *cyp27c1* phylogeny and verify gene identity (Fig. S2).

### Blenny phylogeny and phylogenetic comparative analyses

Because no phylogeny incorporating all our focal blenny species was available from the literature, we created a new phylogeny based on a subset of the transcripts from the retinal transcriptomes (Fig. 1). In detail, we used a reference set of BUSCO genes (in total, 3,507 conserved single-copy genes) extracted from the whole genome of an ovalentarian species, the Nile tilapia, *Oreochromis niloticus* (cichlids; GenBank accession number GCA_001858045.3). We used Geneious v.9.1 with low- to medium-sensitivity settings to map each blenny transcriptome against the tilapia BUSCO gene reference. In total, we mapped 19 blenny samples (two individuals per species/population, except for the single individual for *S. fasciatus*) and an outgroup, the dusky dottyback (*Ps. fuscus*; SRX5060723). We also used the Nile tilapia as an outgroup reference for the phylogenetic analysis. We considered only transcripts with >100 mapped reads and exported the sample-specific consensus sequence for each gene. We then combined the consensuses from different individuals, and only genes found in 17 or more individuals were kept for the subsequent analysis. Next, we aligned each specific gene from all species using MAFFT v.7.407 (Katoh and Standley, 2013). We then concatenated the final set of 338 single-gene alignments, resulting in an 869,725 bp dataset, which we further filtered to contain only sites missing <20% of sequences. The final dataset consisted of 338,152 bp. We reconstructed a phylogenetic tree using Bayesian inference in MrBayes v.3.2.6 (Ronquist et al., 2012), running the analysis for 10 million generations and a burn-in phase of 25% on the CIPRES portal (Miller et al., 2010) and maximum likelihood in RAXML (Stamatakis, 2014) with an automatically determined number of bootstrap replicates. We also tested more stringent filtering to include only genes found in all 21 individuals and in 19 individuals; these datasets comprised 99 (244,332 bp) and 204 (492,296 bp) genes, respectively. These two datasets were then trimmed to exclude sites with more than 5% or 10% missing data (missing in more than one or two species), resulting in two final datasets with 121,722 and 157,917 bp, respectively. The tree topologies from all three datasets and both Bayesian and maximum likelihood analyses were identical (Fig. 1 and UQ eSpace, https://doi.org/10.48610/09af62a). The resulting phylogram was then transformed into an ultrametric tree for use in a PGLS analysis, using the chronoMPL function from the ape v.5.5 package (Paradis and Schliep, 2019).

To compare if the habitat [subtidal (aquatic) versus intertidal (amphibious and terrestrial)] correlates with opsin gene expression in blennies, we used a PGLS using the CAPER package (Orme et al., 2018; https://CRAN.R-project.org/package=caper) in R v.4.1.0 (R Core Team, 2021; https://www.R-project.org/), run through the RStudio interface v.2022.07.2 (R Studio Team, 2022; http://www.rstudio.com/). The PGLS accounts for phylogenetic relationships amongst species by estimating the maximum likelihood of a phylogenetic scaling factor, Pagel's λ, with λ ranging from 0 to 1, representing a continuum from no phylogenetic dependence to absolute phylogenetic dependence. A Bonferroni correction was used to account for multiple testing to adjust significance levels. Only the opsin genes expressed in all species, i.e. *sws2a* paralogues, *rh2a-1*, and *lws*, were analysed, as the sample size was too small for the model to meet basic statistical assumptions otherwise (Table S2).

### Opsin gene sequence analyses

The newly generated blenny phylogeny was used to test for site-specific positive selection on opsin genes using CODEML in PAML (Yang, 2007), as described in detail by Hofmann et al. (2012). When more than one individual of a species was present, we used the consensus nucleotide sequence for this analysis; no intraspecific difference in amino acid sequences was found. Briefly, CODEML was run via the graphical user interface PAMLX v.1.3.1 (Xu and Yang, 2013) using likelihood-ratio tests (LRTs) to compare M1a versus M2 and M8 versus M8a. These models allow the *d*_N_/*d*_S_ ratios to vary amongst codon sites within proteins. M1a and M8 are the null models, in which some sites are under purifying selection (*d*_N_/*d*_S_<1) and others are neutral (*d*_N_/*d*_S_=1). M2 and M8a introduce an additional category, enabling sites to be under positive selection (*d*_N_/*d*_S_>1; Yang, 2007). LRTs identified sites under positive selection by comparing twice the difference in log-likelihood (2Δℓ) between model comparisons with critical values for 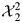 of 5.99 (*P*>0.95) for M1a versus M2 and 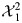 of 2.71 (*P*>0.95) for M8 versus M8a. Bayesian empirical Bayes criteria (Yang et al., 2005) were subsequently applied to identify specific amino acid sites under positive selection in cases with significant LRTs (Table S3).

Blenny opsin amino acid sequences were then aligned to bovine rhodopsin (Palczewski et al., 2000) to assess their variability within the transmembrane and the retinal binding pocket sites, as well as at known opsin spectral tuning sites (Fasick and Robinson, 1998; Hunt et al., 2001; Yokoyama et al., 2007; Yokoyama, 2008; Dungan et al., 2016, reviewed in Hagen et al., 2023). Amino acid substitutions were assessed for each opsin gene, with specific attention to sites that differ in polarity or charge between species (as per Hofmann et al., 2012) (Table S4).

## Supplementary Material



10.1242/biolopen.062593_sup1 Supplementary information

Table S4. Opsin amino acid comparisons.

## References

[BIO062593C1] Afgan, E., Sloggett, C., Goonasekera, N., Makunin, I., Benson, D., Crowe, M., Gladman, S., Kowsar, Y., Pheasant, M. and Horst, R. (2015). Genomics virtual laboratory: a practical bioinformatics workbench for the cloud. *PLoS ONE* 10, e0140829. 10.1371/journal.pone.0140829 26501966 PMC4621043

[BIO062593C2] Alfaro, M. E., Faircloth, B. C., Harrington, R. C., Sorenson, L., Friedman, M., Thacker, C. E., Oliveros, C. H., Černý, D. and Near, T. J. (2018). Explosive diversification of marine fishes at the Cretaceous–Palaeogene boundary. *Nat. Ecol. Evol.* 2, 688-696. 10.1038/s41559-018-0494-6 29531346

[BIO062593C3] Baden, T., Angueyra, J. M., Bosten, J. M., Collin, S. P., Conway, B., Cortesi, F., Dedek, K., Euler, T., Novales Flamarique, I., Franklin, A. et al. (2025). A standardized nomenclature for the rods and cones of the vertebrate retina. *PLoS Genet.* 23, e3003157. 10.1371/journal.pbio.3003157 PMC12057980 40333813

[BIO062593C4] Bhikajee, M. and Green, J. M. (2002). Behaviour and habitat of the Indian Ocean amphibious blenny, *Alticus monochrus*. *Afr. Zool.* 37, 221-230. 10.1080/15627020.2002.11657177

[BIO062593C5] Bolger, A. M., Lohse, M. and Usadel, B. (2014). Trimmomatic: a flexible trimmer for Illumina sequence data. *Bioinformatics* 30, 2114-2120. 10.1093/bioinformatics/btu170 24695404 PMC4103590

[BIO062593C7] Burguera, D., Dionigi, F., Kverková, K., Winkler, S., Brown, T., Pippel, M., Zhang, Y., Shafer, M., Nichols, A. L. A., Myers, E. et al. (2023). Expanded olfactory system in ray-finned fishes capable of terrestrial exploration. *BMC Biol.* 21, 163. 10.1186/s12915-023-01661-8 37525196 PMC10392011

[BIO062593C8] Carleton, K. L., Spady, T. C., Streelman, J. T., Kidd, M. R., McFarland, W. N. and Loew, E. R. (2008). Visual sensitivities tuned by heterochronic shifts in opsin gene expression. *BMC Biol.* 6, 22. 10.1186/1741-7007-6-22 18500997 PMC2430543

[BIO062593C9] Carleton, K. L., Escobar-Camacho, D., Stieb, S. M., Cortesi, F. and Marshall, N. J. (2020). Seeing the rainbow: mechanisms underlying spectral sensitivity in teleost fishes. *J. Exp. Biol.* 223, jeb193334. 10.1242/jeb.193334 32327561 PMC7188444

[BIO062593C10] Cheney, K. L., Skogh, C., Hart, N. S. and Marshall, N. J. (2009). Mimicry, colour forms and spectral sensitivity of the bluestriped fangblenny, *Plagiotremus rhinorhynchos*. *Proc. R. Soc. B* 276, 1565-1573. 10.1098/rspb.2008.1819 PMC2660993 19324827

[BIO062593C12] Cortesi, F., Musilová, Z., Stieb, S. M., Hart, N. S., Siebeck, U. E., Malmstrøm, M., Tørresen, O. K., Jentoft, S., Cheney, K. L., Marshall, N. J. et al. (2015). Ancestral duplications and highly dynamic opsin gene evolution in percomorph fishes. *Proc. Natl Acad. Sci. USA* 112, 1493-1498. 10.1073/pnas.1417803112 25548152 PMC4321309

[BIO062593C13] Cortesi, F., Musilová, Z., Stieb, S. M., Hart, N. S., Siebeck, U. E., Cheney, K. L., Salzburger, W. and Marshall, N. J. (2016). From crypsis to mimicry: changes in colour and the configuration of the visual system during ontogenetic habitat transitions in a coral reef fish. *J. Exp. Biol.* 219, 2545-2558. 10.1242/jeb.139501 27307489

[BIO062593C14] Cortesi, F., Camacho, D. E., Sommer, G. M., Luehrmann, M., Hidber, F., Deupi, X., Carleton, K. and Musilova, Z. (2021). Turning red to green repeatedly: ancient LWS duplications drive convergent visual tuning in teleost fishes. *bioRxiv* 2021.05.08.443214. 10.1101/2021.05.08.443214

[BIO062593C16] Dalton, B. E., de Busserolles, F., Marshall, N. J. and Carleton, K. L. (2017). Retinal specialization through spatially varying cell densities and opsin coexpression in cichlid fish. *J. Exp. Biol.* 220, 266-277. 10.1242/jeb.149211 27811302 PMC6514458

[BIO062593C17] Darriba, D., Taboada, G. L., Doallo, R. and Posada, D. (2012). jModelTest 2: more models, new heuristics and parallel computing. *Nat. Methods* 9, 772. 10.1038/nmeth.2109 PMC4594756 22847109

[BIO062593C18] de Busserolles, F., Cortesi, F., Helvik, J. V., Davies, W. I., Templin, R. M., Sullivan, R. K., Michell, C. T., Mountford, J. K., Collin, S. P., Irigoien, X. et al. (2017). Pushing the limits of photoreception in twilight conditions: the rod-like cone retina of the deep-sea pearlsides. *Sci. Adv.* 3, eaao4709. 10.1126/sciadv.aao4709 29134201 PMC5677336

[BIO062593C19] Dungan, S. Z., Kosyakov, A. and Chang, B. S. W. (2016). Spectral tuning of killer whale (*Orcinus orca*) rhodopsin: evidence for positive selection and functional adaptation in a cetacean visual pigment. *Mol. Biol. Evol.* 33, 323-336. 10.1093/molbev/msv217 26486871

[BIO062593C20] Enright, J. M., Toomey, M. B., Sato, S.-Y., Temple, S. E., Allen, J. R., Fujiwara, R., Kramlinger, V. M., Nagy, L. D., Johnson, K. M., Xiao, Y. et al. (2015). Cyp27c1 red-shifts the spectral sensitivity of photoreceptors by converting vitamin A1 into A2. *Curr. Biol.* 25, 3048-3057. 10.1016/j.cub.2015.10.018 26549260 PMC4910640

[BIO062593C21] Escobar-Camacho, D., Ramos, E., Martins, C. and Carleton, K. L. (2017). The opsin genes of Amazonian cichlids. *Mol. Ecol.* 26, 1343-1356. 10.1111/mec.13957 PMC5342946 27997048

[BIO062593C22] Fasick, J. I. and Robinson, P. R. (1998). Mechanism of spectral tuning in the dolphin visual pigments. *Biochemistry* 37, 433-438. 10.1021/bi972500j 9471225

[BIO062593C23] Fuller, R. C., Carleton, K. L., Fadool, J. M., Spady, T. C. and Travis, J. (2005). Genetic and environmental variation in the visual properties of bluefin killifish, *Lucania goodei*. *J. Evol. Biol.* 18, 516-523. 10.1111/j.1420-9101.2005.00886.x 15842481

[BIO062593C24] Haas, B. J., Papanicolaou, A., Yassour, M., Grabherr, M., Blood, P. D., Bowden, J., Couger, M. B., Eccles, D., Li, B., Lieber, M. et al. (2013). *De novo* transcript sequence reconstruction from RNA-seq using the Trinity platform for reference generation and analysis. *Nat. Protoc.* 8, 1494. 10.1038/nprot.2013.084 23845962 PMC3875132

[BIO062593C25] Hagen, J. F., Roberts, N. S. and Johnston, R. J. Jr. (2023). The evolutionary history and spectral tuning of vertebrate visual opsins. *Dev. Biol.* 493, 40-66. 10.1016/j.ydbio.2022.10.014 36370769 PMC9729497

[BIO062593C26] Härer, A., Meyer, A. and Torres-Dowdall, J. (2018). Convergent phenotypic evolution of the visual system via different molecular routes: how neotropical cichlid fishes adapt to novel light environments. *Evol. Lett.* 2, 341-354. 10.1002/evl3.71

[BIO062593C27] Hofmann, C. M., O'Quin, K. E., Marshall, N. J., Cronin, T. W., Seehausen, O. and Carleton, K. L. (2009). The eyes have it: regulatory and structural changes both underlie cichlid visual pigment diversity. *PLoS Biol.* 7, e1000266. 10.1371/journal.pbio.1000266 20027211 PMC2790343

[BIO062593C28] Hofmann, C. M., Marshall, N. J., Abdilleh, K., Patel, Z., Siebeck, U. E. and Carleton, K. L. (2012). Opsin evolution in damselfish: convergence, reversal, and parallel evolution across tuning sites. *J. Mol. Evol.* 75, 79-91. 10.1007/s00239-012-9525-0 23080353

[BIO062593C29] Hsieh, S.-T. T. (2010). A locomotor innovation enables water-land transition in a marine fish. *PLoS ONE* 5, e11197. 10.1371/journal.pone.0011197 20585564 PMC2887833

[BIO062593C30] Hundt, P. J. and Simons, A. M. (2018). Extreme dentition does not prevent diet and tooth diversification within combtooth blennies (Ovalentaria: Blenniidae). *Evolution* 72, 930-943. 10.1111/evo.13453 29457222

[BIO062593C31] Hundt, P. J., Iglésias, S. P., Hoey, A. S. and Simons, A. M. (2014a). A multilocus molecular phylogeny of combtooth blennies (Percomorpha: Blennioidei: Blenniidae): multiple invasions of intertidal habitats. *Mol. Phylogenet. Evol.* 70, 47-56. 10.1016/j.ympev.2013.09.001 24045103

[BIO062593C32] Hundt, P. J., Nakamura, Y. and Yamaoka, K. (2014b). Diet of combtooth blennies (Blenniidae) in Kochi and Okinawa, Japan. *Ichthyol. Res.* 61, 76-82. 10.1007/s10228-013-0366-7

[BIO062593C33] Hunt, D. M., Dulai, K. S., Partridge, J. C., Cottrill, P. and Bowmaker, J. K. (2001). The molecular basis for spectral tuning of rod visual pigments in deep-sea fish. *J. Exp. Biol.* 204, 3333-3344. 10.1242/jeb.204.19.3333 11606607

[BIO062593C34] Hunt, D. M., Hankins, M. W., Collin, S. P. and Marshall, N. J. (2014). *Evolution of Visual and Non-Visual Pigments*. Springer.

[BIO062593C35] Ivanov, I. V., Mappes, T., Schaupp, P., Lappe, C. and Wahl, S. (2018). Ultraviolet radiation oxidative stress affects eye health. *J. Biophotonics* 11, e201700377. 10.1002/jbio.201700377 29603665

[BIO062593C36] Katoh, K. and Standley, D. M. (2013). MAFFT multiple sequence alignment software version 7: improvements in performance and usability. *Mol. Biol. Evol.* 30, 772-780. 10.1093/molbev/mst010 23329690 PMC3603318

[BIO062593C38] Korenbrot, J. I. and Fernald, R. D. (1989). Circadian rhythm and light regulate opsin mRNA in rod photoreceptors. *Nature* 337, 454-457. 10.1038/337454a0 2521689

[BIO062593C40] Lin, J.-J., Wang, F.-Y., Li, W.-H. and Wang, T.-Y. (2017). The rises and falls of opsin genes in 59 ray-finned fish genomes and their implications for environmental adaptation. *Sci. Rep.* 7, 15568. 10.1038/s41598-017-15868-7 29138475 PMC5686071

[BIO062593C93] Loew, E. R. and Lythgoe, J. N. (1978). The ecology of cone pigments in teleost fishes. *Vis. Res.* 18, 715-722. 10.1016/0042-6989(78)90150-5 664357

[BIO062593C41] Luehrmann, M., Cortesi, F., Cheney, K. L., de Busserolles, F. and Marshall, N. J. (2020). Microhabitat partitioning correlates with opsin gene expression in coral reef cardinalfishes (Apogonidae). *Funct. Ecol.* 34, 1041-1052. 10.1111/1365-2435.13529

[BIO062593C42] MacIver, M. A., Schmitz, L., Mugan, U., Murphey, T. D. and Mobley, C. D. (2017). Massive increase in visual range preceded the origin of terrestrial vertebrates. *Proc. Natl Acad. Sci. USA* 114, E2374-E2384. 10.1073/pnas.1615563114 PMC5373340 28270619

[BIO062593C43] Marshall, N. J., Cortesi, F., de Busserolles, F., Siebeck, U. E. and Cheney, K. L. (2018). Colours and colour vision in reef fishes: past, present and future research directions. *J. Fish Biol.* 95, 5-38. 10.1111/jfb.13849 30357835

[BIO062593C44] Miller, M. A., Pfeiffer, W. and Schwartz, T. (2010). Creating the CIPRES Science Gateway for inference of large phylogenetic trees. 2010 Gateway Computing Environments Workshop (GCE), New Orleans, LA, USA, 2010, pp. 1-8, 10.1109/GCE.2010.5676129.

[BIO062593C45] Morgans, C. L. and Ord, T. J. (2013). Natural selection in novel environments: predation selects for background matching in the body colour of a land fish. *Anim. Behav.* 86, 1241-1249. 10.1016/j.anbehav.2013.09.027

[BIO062593C46] Morgans, C. L., Cooke, G. M. and Ord, T. J. (2014). How populations differentiate despite gene flow: sexual and natural selection drive phenotypic divergence within a land fish, the Pacific leaping blenny. *BMC Evol. Biol.* 14, 97. 10.1186/1471-2148-14-97 24884492 PMC4055934

[BIO062593C47] Musilova, Z. and Cortesi, F. (2023). The evolution of the green-light-sensitive visual opsin genes (*RH2*) in teleost fishes. *Vision Res.* 206, 108204. 10.1016/j.visres.2023.108204 36868011

[BIO062593C48] Musilova, Z., Cortesi, F., Matschiner, M., Davies, W. I. L., Patel, J. S., Stieb, S. M., de Busserolles, F., Malmstrøm, M., Tørresen, O. K., Brown, C. J. et al. (2019). Vision using multiple distinct rod opsins in deep-sea fishes. *Science* 364, 588-592. 10.1126/science.aav4632 31073066 PMC6628886

[BIO062593C94] Musilova, Z., Salzburger, W. and Cortesi, F. (2021). The visual opsin gene repertoires of teleost fishes: evolution, ecology, and function. *Annu. Rev. Cell Dev. Biol.* 37, 441-468. 10.1146/annurev-cellbio-120219-024915 34351785

[BIO062593C49] Ord, T. J. and Cooke, G. M. (2016). Repeated evolution of amphibious behavior in fish and its implications for the colonization of novel environments. *Evolution* 70, 1747-1759. 10.1111/evo.12971 27272014

[BIO062593C50] Ord, T. J. and Hsieh, S. T. (2011). A highly social, land-dwelling fish defends territories in a constantly fluctuating environment. *Ethology* 117, 918-927. 10.1111/j.1439-0310.2011.01949.x

[BIO062593C51] Ord, T. J. and Hundt, P. J. (2020). Crossing extreme habitat boundaries: jack-of-all-trades facilitates invasion but is eroded by adaptation to a master-of-one. *Funct. Ecol.* 34, 1404-1415. 10.1111/1365-2435.13600

[BIO062593C52] Ord, T. J., Summers, T. C., Noble, M. M. and Fulton, C. J. (2017). Ecological release from aquatic predation is associated with the emergence of marine blenny fishes onto land. *Am. Nat.* 189, 570-579. 10.1086/691155 28410030

[BIO062593C53] Palczewski, K., Kumasaka, T., Hori, T., Behnke, C. A., Motoshima, H., Fox, B. A., Le Trong, I., Teller, D. C., Okada, T., Stenkamp, R. E. et al. (2000). Crystal structure of rhodopsin: a G protein-coupled receptor. *Science* 289, 739-745. 10.1126/science.289.5480.739 10926528

[BIO062593C54] Paradis, E. and Schliep, K. (2019). ape 5.0: an environment for modern phylogenetics and evolutionary analyses in R. *Bioinformatics* 35, 526-528. 10.1093/bioinformatics/bty633 30016406

[BIO062593C55] Platt, E. R. and Ord, T. J. (2015). Population variation in the life history of a land fish, *Alticus arnoldorum*, and the effects of predation and density. *PLoS ONE* 10, e0137244. 10.1371/journal.pone.0137244 26398191 PMC4580579

[BIO062593C56] Policarpo, M., Fogg, L. G., Cortesi, F. and Salzburger, W. (2025). Evolution of the non-visual and visual opsin gene repertoire in ray-finned fishes. *Genome Biol. Evol.* 17, evaf129. 10.1093/gbe/evaf129 40576097 PMC12279438

[BIO062593C57] Rodd, F. H., Hughes Kimberly, A., Grether Gregory, F. and Baril Colette, T. (2002). A possible non-sexual origin of mate preference: are male guppies mimicking fruit? *Proc. R. Soc. B* 269, 475-481. 10.1098/rspb.2001.1891 PMC1690917 11886639

[BIO062593C58] Ronquist, F., Teslenko, M., van der Mark, P., Ayres, D. L., Darling, A., Höhna, S., Larget, B., Liu, L., Suchard, M. A. and Huelsenbeck, J. P. (2012). MrBayes 3.2: efficient Bayesian phylogenetic inference and model choice across a large model space. *Syst. Biol.* 61, 539-542. 10.1093/sysbio/sys029 22357727 PMC3329765

[BIO062593C60] Sandkam, B., Dalton, B., Breden, F., Carleton, K. L. and Fuller, H. E. B. (2018). Reviewing guppy color vision: integrating the molecular and physiological variation in visual tuning of a classic system for sensory drive. *Curr. Zool.* 64, 535-545. 10.1093/cz/zoy047 30108634 PMC6084590

[BIO062593C61] Savelli, I., Flamarique, I. N., Iwanicki, T. and Taylor, J. S. (2018). Parallel opsin switches in multiple cone types of the starry flounder retina: tuning visual pigment composition for a demersal life style. *Sci. Rep.* 8, 4763. 10.1038/s41598-018-23008-y 29555918 PMC5859124

[BIO062593C62] Sayer, M. D. (2005). Adaptations of amphibious fish for surviving life out of water. *Fish Fish.* 6, 186-211. 10.1111/j.1467-2979.2005.00193.x

[BIO062593C63] Schweikert, L. E., Fitak, R. R., Caves, E. M., Sutton, T. T. and Johnsen, S. (2018). Spectral sensitivity in ray-finned fishes: diversity, ecology and shared descent. *J. Exp. Biol.* 221, jeb189761. 10.1242/jeb.189761 30322978

[BIO062593C64] Seehausen, O., Terai, Y., Magalhaes, I. S., Carleton, K. L., Mrosso, H. D., Miyagi, R., van der Sluijs, I., Schneider, M. V., Maan, M. E., Tachida, H. et al. (2008). Speciation through sensory drive in cichlid fish. *Nature* 455, 620-626. 10.1038/nature07285 18833272

[BIO062593C65] Shaughnessy, A. and Cortesi, F. (2025). Coral reef fish visual adaptations to a changing world. *Funct. Ecol.* 39, 2561-2572. 10.1111/1365-2435.14668

[BIO062593C66] Shimizu, N., Sakai, Y., Hashimoto, H. and Gushima, K. (2006). Terrestrial reproduction by the air-breathing fish *Andamia tetradactyla* (Pisces; Blenniidae) on supralittoral reefs. *J. Zool.* 269, 357-364. 10.1111/j.1469-7998.2006.00113.x

[BIO062593C67] Siebeck, U. E. and Marshall, N. J. (2007). Potential ultraviolet vision in pre-settlement larvae and settled reef fish – a comparison across 23 families. *Vision Res.* 47, 2337-2352. 10.1016/j.visres.2007.05.014 17632200

[BIO062593C68] Smith, C., Barber, I., Wootton Robert, J. and Chittka, L. (2004). A receiver bias in the origin of three-spined stickleback mate choice. *Proc. R. Soc. B* 271, 949-955. 10.1098/rspb.2004.2690 PMC1691680 15255050

[BIO062593C69] Spady, T. C., Parry, J. W., Robinson, P. R., Hunt, D. M., Bowmaker, J. K. and Carleton, K. L. (2006). Evolution of the cichlid visual palette through ontogenetic subfunctionalization of the opsin gene arrays. *Mol. Biol. Evol.* 23, 1538-1547. 10.1093/molbev/msl014 16720697

[BIO062593C70] Stamatakis, A. (2014). RAxML version 8: a tool for phylogenetic analysis and post-analysis of large phylogenies. *Bioinformatics* 30, 1312-1313. 10.1093/bioinformatics/btu033 24451623 PMC3998144

[BIO062593C71] Stieb, S. M., Carleton, K. L., Cortesi, F., Marshall, N. J. and Salzburger, W. (2016). Depth-dependent plasticity in opsin gene expression varies between damselfish (Pomacentridae) species. *Mol. Ecol.* 25, 3645-3661. 10.1111/mec.13712 27262029

[BIO062593C72] Stieb, S. M., Cortesi, F., Sueess, L., Carleton, K. L., Salzburger, W. and Marshall, N. J. (2017). Why UV vision and red vision are important for damselfish (Pomacentridae): structural and expression variation in opsin genes. *Mol. Ecol.* 26, 1323-1342. 10.1111/mec.13968 27997050

[BIO062593C73] Stieb, S. M., de Busserolles, F., Carleton, K. L., Cortesi, F., Chung, W. S., Dalton, B., Hammond, L. A. and Marshall, N. J. (2019). A detailed investigation of the visual system and visual ecology of the barrier reef anemonefish, *Amphiprion akindynos*. *Sci. Rep.* 9, 16459. 10.1038/s41598-019-52297-0 PMC6848076 31712572

[BIO062593C74] Stieb, S. M., Cortesi, F., Jardim De Queiroz, L., Carleton, K. L., Seehausen, O. and Marshall, N. J. (2023). Long-wavelength-sensitive (*lws*) opsin gene expression, foraging and visual communication in coral reef fishes. *Mol. Ecol.* 32, 1656-1672. 10.1111/mec.16831 36560895 PMC10065935

[BIO062593C76] Swamynathan, S. K., Crawford, M., Robison JR, W. G., Kanungo, J. and Piatigorsky, J. (2003). Adaptive differences in the structure and macromolecular compositions of the air and water corneas of the ‘four-eyed’ fish (*Anableps anableps*). *FASEB J.* 17, 1996-2005. 10.1096/fj.03-0122com 14597669 PMC5998667

[BIO062593C77] Tettamanti, V., de Busserolles, F., Lecchini, D., Marshall, N. J. and Cortesi, F. (2019). Visual system development of the spotted unicornfish, *Naso brevirostris* (Acanthuridae). *J. Exp. Biol.* 222, jeb209916. 10.1242/jeb.209916 31776185

[BIO062593C79] Wald, G. (1968). The molecular basis of visual excitation. *Nature* 219, 800-807. 10.1038/219800a0 4876934

[BIO062593C80] White, E. M., Goncalves, D. M., Partridge, J. C. and Oliveira, R. F. (2004). Vision and visual variation in the peacock blenny. *J. Fish Biol.* 65, 227-250. 10.1111/j.0022-1112.2004.00446.x

[BIO062593C81] Wright, P. A. and Turko, A. J. (2016). Amphibious fishes: evolution and phenotypic plasticity. *J. Exp. Biol.* 219, 2245-2259. 10.1242/jeb.126649 27489213

[BIO062593C82] Xu, B. and Yang, Z. (2013). PAMLX: a graphical user interface for PAML. *Mol. Biol. Evol.* 30, 2723-2724. 10.1093/molbev/mst179 24105918

[BIO062593C83] Yang, Z. (2007). PAML 4: phylogenetic analysis by maximum likelihood. *Mol. Biol. Evol.* 24, 1586-1591. 10.1093/molbev/msm088 17483113

[BIO062593C84] Yang, Z., Wong, W. S. and Nielsen, R. (2005). Bayes empirical Bayes inference of amino acid sites under positive selection. *Mol. Biol. Evol.* 22, 1107-1118. 10.1093/molbev/msi097 15689528

[BIO062593C85] Yao, S. and Ord, T. J. (2025). Adaptation for crypsis versus conspicuous social signalling following transitions across an extreme ecotone. *J. Evol. Biol.* 38, 580-593. 10.1093/jeb/voaf025 40109254

[BIO062593C86] Yokoyama, S. (2002). Molecular evolution of color vision in vertebrates. *Gene* 300, 69-78. 10.1016/S0378-1119(02)00845-4 12468088

[BIO062593C87] Yokoyama, S. (2008). Evolution of dim-light and color vision pigments. *Annu. Rev. Genomics Hum. Genet.* 9, 259-282. 10.1146/annurev.genom.9.081307.164228 18544031

[BIO062593C88] Yokoyama, S., Takenaka, N. and Blow, N. (2007). A novel spectral tuning in the short wavelength-sensitive (SWS1 and SWS2) pigments of bluefin killifish (*Lucania goodei*). *Gene* 396, 196-202. 10.1016/j.gene.2007.03.019 17498892 PMC1963460

[BIO062593C89] You, X., Bian, C., Zan, Q., Xu, X., Liu, X., Chen, J., Wang, J., Qiu, Y., Li, W., Zhang, X. et al. (2014). Mudskipper genomes provide insights into the terrestrial adaptation of amphibious fishes. *Nat. Commun.* 5, 5594. 10.1038/ncomms6594 25463417 PMC4268706

[BIO062593C90] Yourick, M. R., Sandkam, B. A., Gammerdinger, W. J., Escobar-Camacho, D., Nandamuri, S. P., Clark, F. E., Joyce, B., Conte, M. A., Kocher, T. D. and Carleton, K. L. (2019). Diurnal variation in opsin expression and common housekeeping genes necessitates comprehensive normalization methods for quantitative real-time PCR analyses. *Mol. Ecol. Resources* 19, 1447-1460. 10.1111/1755-0998.13062 PMC6995727 31325910

[BIO062593C91] Zagarese, H. E. and Williamson, C. E. (2001). The implications of solar UV radiation exposure for fish and fisheries. *Fish Fish.* 2, 250-260. 10.1046/j.1467-2960.2001.00048.x

[BIO062593C92] Zander, C. D. (1974). Beziehungen zwischen Körperbau und Lebensweise bei Blenniidae (Pisces) aus dem Roten Meer. III. Morphologie des Auges. *Marine Biology* 28, 61-71. 10.1007/BF00389118

