# Supplementary material for: Opsin gene evolution in aquatic, amphibious and terrestrial blennies (Blenniidae)
Source: Biol Open. 2026 Jul 15;15(7):bio062593. doi: 10.1242/bio.062593 (PMC13446567; doi:10.1242/bio.062593)
Supplement: Supplementary file 1 [file biolopen-15-062593-s1.pdf]

Table S1. GenBank accession numbers for the molecular data produced during this study. *rh1* = rod opsin, *sws* = short-wavelength-sensitive, *rh2* = rhodopsin-like 2, *lws* = long-wavelength-sensitive, *cyp27c1* = cytochrome P450 family 27 subfamily c member 1. *data from Stieb et al. (2023) and **Musilova et al. (2019).

Species	***GenBank accession no.***	***Gene/Transcriptome ID***
Alticus simplicirrus	PZ509123	*rh1*
	PZ509124	*rh2a-1*
	PZ509125	*rh2a-2*
	PZ509107	*sws2aα*
	PZ509108	*sws2aβ*
	PZ509122	*lws*
	PZ509098	*cyp27c1*
	SAMN60550786	B7
	SAMN60550787	B8
	SAMN60550788	B9
Alticus anjouanae	PZ509120	*rh1*
	PZ509121	*rh2a-1*
	PZ509105	*sws2aα*
	PZ509106	*sws2aβ*
	PZ509119	*lws*
	PZ509097	*cyp27c1*
	SAMN60550789	B10
	SAMN60550790	B11
	SAMN60550791	B12
Praealticus caesius	PZ509139	*rh1*
	PZ509140	*rh2a-1*
	partial – available on request	*rh2a-2*
	PZ509115	*sws2aα*
	PZ509116	*sws2aβ*
	PZ509138	*lws*

Biology Open • Supplementary information

	PZ509099	*cyp27c1*
	SAMN60550792	B4
	SAMN60550793	B5
	SAMN60550794	B6
Entomacrodus striatus	PZ509128	*rh1*
	PZ509129	*rh2a-1*
	PZ509109	*sws2aα*
	PZ509110	*sws2aβ*
	PZ509127	*lws*
	PZ509100	*cyp27c1*
	SAMN60550795	B13
	SAMN60550796	B14
	SAMN60550797	B15
Istiblennius lineatus	PZ509131	*rh1*
	PZ509132	*rh2a-1*
	PZ509133	*rh2a-2*
	PZ509111	*sws2aα*
	PZ509112	*sws2aβ*
	PZ509130	*lws*
	PZ509101	*cyp27c1*
	SAMN60550798	B1
	SAMN60550799	B2
	SAMN60550800	B3
	SAMN60550801	B16
	SAMN60550802	B17
	SAMN60550803	B18
Salarias fasciatus	PZ509142	*rh1*
	PZ509143	*rh2a-1*
	PZ509144	*rh2b*
	PZ509117	*sws2aα*
	PZ509118	*sws2aβ*
	PZ509141	*lws*

	PZ509102	*cyp27c1*
	SAMN60550804	SF1HRS2017
Ecsenius bicolor	OK350547*	*rh1*
	OK350548*	*rh2a-1*
	OK350549*/ PZ509126	*rh2b*
	OK350551*	*sws2aα*
	OK350552*	*sws2aβ*
	OK350550*	*sws1*
	OK350546*	*lws*
	PZ509103	*cyp27c1*
	SRX12410465*	FB10
	SRX12410464*	FB2
	SRX12410466*	C4
Meiacanthus atrodorsalis	OK350564*	*rh1*
	OK350565*	*rh2a-1*
	OK350566*	*rh2b*
	OK350568*	*sws2aα*
	OK350569*	*sws2aβ*
	OK350567*	*sws1*
	OK350563*	*lws*
	PZ509104	*cyp27c1*
	SRX12410468*	FD9
	SRX12410469*	GA1
	SRX12410467*	S209
Plagiotremus rhinorhynchos	PZ509135	*rh1*
	PZ509136	*rh2a-1*
	PZ509137	*rh2b*
	PZ509113	*sws2aα*
	PZ509114	*sws2aβ*
	PZ509134	*lws*
	SRX5060699*	FZ7
	SRX5060700*	FZ8

Biology Open • Supplementary information

Table S2. Summary of the phylogenetic generalised least squares regression (pGLS) comparing opsin gene expression in blennies to their habitat.

Opsin	Categories tested	Pagel's λ (ML)	Adjusted R^2	Residual standard error	Degrees of Freedom	F-statistic	P-value
sws2aα	1. Terrestrial, amphibious, aquatic	0.982	0.431	50.63	2, 5	3.648	0.105
	2. intertidal, subtidal	1	0.047	66.16	1, 6	1.347	0.29
sws2aβ	1. Terrestrial, amphibious, aquatic	0.982	0.431	50.62	2, 5	3.647	0.106
	2. intertidal, subtidal	1	0.047	66.15	1, 6	1.345	0.29
rh2a-1	1. Terrestrial, amphibious, aquatic	0	0.35	13.21	2, 5	2.884	0.147
	2. intertidal, subtidal	0	-0.054	16.81	1, 6	0.641	0.454
lws	1. Terrestrial, amphibious, aquatic	1	-0.019	24.58	2, 5	0.934	0.452
	2. intertidal, subtidal	1	-0.145	26.1	1, 6	0.09	0.775

Biology Open • Supplementary information

Table S3. PAML selection analyses for the blenny opsin genes using the CODEML models M1a *vs.* M2 and M8 *vs.* M8a (Yang 2007). Note that none of the genes was found to be under positive selection.

**M2 versus M1a**						
	**M2 (lnL)**	***ω* for K = 3**	**M1a (lnL)**	***ω* for K = 2**	**−2ΔlnL**	***P***
**RH1**	-2523.29	*P*: 0.92, 0.05, 0.03<br>*ω*: 0.00, 1.00, 1.87	-2524.05	*P*: 0.90, 0.10<br>*ω*: 0.0, 1.00	1.52	*ns*
**SWS2Aα**	-3159.48	*P*: 0.93, 0.05, 0.03<br>*ω*: 0.04, 1.00, 1.00	-3159.48	*P*: 0.93, 0.07<br>*ω*: 0.04, 1.00	0.00	*ns*
**SWS2Aβ**	-2764.56	*P*: 0.92, 0.04, 0.05<br>*ω*: 0.01, 1.00, 1.00	-2764.56	*P*: 0.93, 0.07<br>*ω*: 0.01, 1.00	0.00	*ns*
**RH2A**	-2536.18	*P*: 0.93, 0.06, 0.00<br>*ω*: 0.03, 1.00, 2.45	-2536.22	*P*: 0.92, 0.08<br>*ω*: 0.02, 1.00	0.08	*ns*
**LWS**	-2548.52	*P*: 0.94, 0.06, 0.01<br>*ω*: 0.02, 1.00, 3.25	-2549.07	*P*: 0.93, 0.07<br>*ω*: 0.01, 1.00	1.1	*ns*
**M8 versus M8a**						
	**M8 (lnL)**	***ω* for K = 11**	**M8a (lnL)**	***ω* for K = 11**	**−2ΔlnL**	***P***
**RH1**	-2523.30	*P*: 0.09, 0.09, 0.09, 0.09, 0.09, 0.09, 0.09, 0.09, 0.09, 0.09, 0.07<br>*ω*: 0.00, 0.00, 0.00, 0.00, 0.00, 0.00, 0.00, 0.00, 0.00, 0.10, 1.50	-2524.05	*P*: 0.09, 0.09, 0.09, 0.09, 0.09, 0.09, 0.09, 0.09, 0.09, 0.09, 0.10<br>*ω*: 0.00, 0.00, 0.00, 0.00, 0.00, 0.00, 0.00, 0.00, 0.00, 0.00, 1.00	1.5	*ns*
**SWS2Aα**	-3158.45	*P*: 0.10, 0.10, 0.10, 0.10, 0.10, 0.10, 0.10, 0.10, 0.10, 0.10, 0.03<br>*ω*: 0.00, 0.00, 0.00, 0.00, 0.01, 0.02, 0.04, 0.08, 0.17, 0.38, 1.23	-3158.51	*P*: 0.10, 0.10, 0.10, 0.10, 0.10, 0.10, 0.10, 0.10, 0.10, 0.10, 0.05<br>*ω*: 0.00, 0.00, 0.00, 0.00, 0.01, 0.02, 0.04, 0.07, 0.14, 0.31, 1.00	0.12	*ns*
**SWS2Aβ**	-2763.42	*P*: 0.10, 0.10, 0.10, 0.10, 0.10, 0.10, 0.10, 0.10, 0.10, 0.10, 0.02<br>*ω*: 0.00, 0.00, 0.00, 0.00, 0.00, 0.00, 0.00, 0.00, 0.01, 0.58, 1.00	-2763.42	*P*: 0.10, 0.10, 0.10, 0.10, 0.10, 0.10, 0.10, 0.10, 0.10, 0.10, 0.02<br>*ω*: 0.00, 0.00, 0.00, 0.00, 0.00, 0.00, 0.00, 0.00, 0.00, 0.58, 1.00	0.00	*ns*
**RH2A**	-2535.99	*P*: 0.10, 0.10, 0.10, 0.10, 0.10, 0.10, 0.10, 0.10, 0.10, 0.10, 0.01<br>*ω*: 0.00, 0.00, 0.00, 0.00, 0.00, 0.00, 0.00, 0.02, 0.12, 0.51, 1.96	-2536.19	*P*: 0.10, 0.10, 0.10, 0.10, 0.10, 0.10, 0.10, 0.10, 0.10, 0.10, 0.06<br>*ω*: 0.00, 0.00, 0.00, 0.00, 0.00, 0.00, 0.01, 0.03, 0.07, 0.18, 1.00	0.40	*ns*

Biology Open • Supplementary information

**LWS**	-2548.57	*P*: 0.10, 0.10, 0.10, 0.10, 0.10, 0.10, 0.10, 0.10, 0.10, 0.10, 0.02<br>*ω*: 0.00, 0.00, 0.00, 0.00, 0.00, 0.00, 0.00, 0.00, 0.04, 0.44, 2.13	-2549.08	*P*: 0.09, 0.09, 0.09, 0.09, 0.09, 0.09, 0.09, 0.09, 0.09, 0.09, 0.07<br>*ω*: 0.00, 0.01, 0.01, 0.01, 0.01, 0.01, 0.02, 0.02, 0.02, 0.04, 1.00	1.02	*ns*

ns, non-significant

Table S4. Opsin amino acid comparisons.

Available for download at
https://journals.biologists.com/bio/article-lookup/doi/10.1242/bio.062593#supplementary-data

Biology Open • Supplementary information

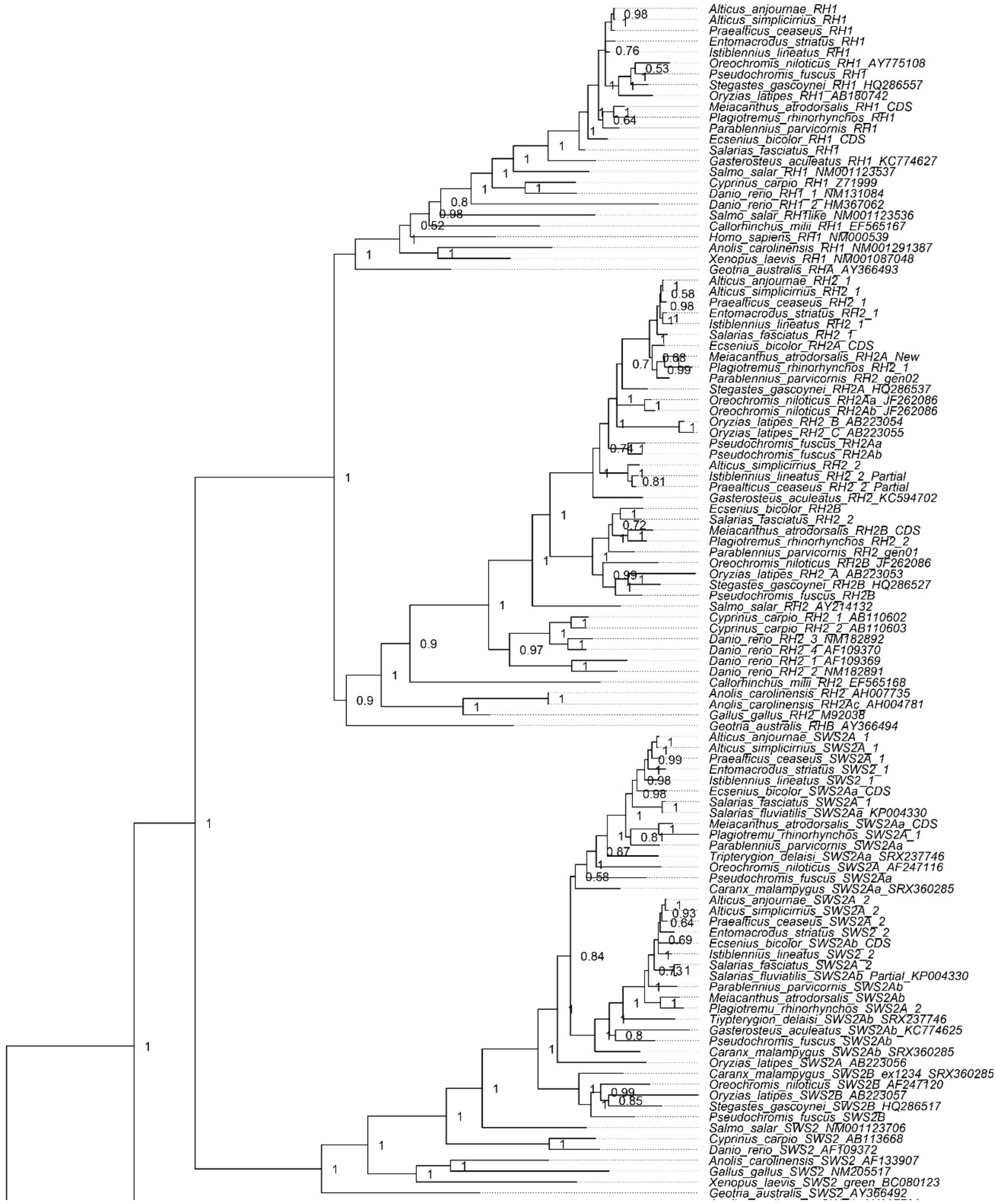

Fig. S1. Bayesian consensus phylogeny of vertebrate visual opsin gene coding regions. *rh1* = rod opsin, *sws* = short-wavelength-sensitive, *rh2* = rhodopsin-like 2, *lws* = long-wavelength-sensitive, *va* = vertebrate ancestral opsin. Bayesian posterior probabilities are inset in bold. Continued on next page.

Biology Open • Supplementary information

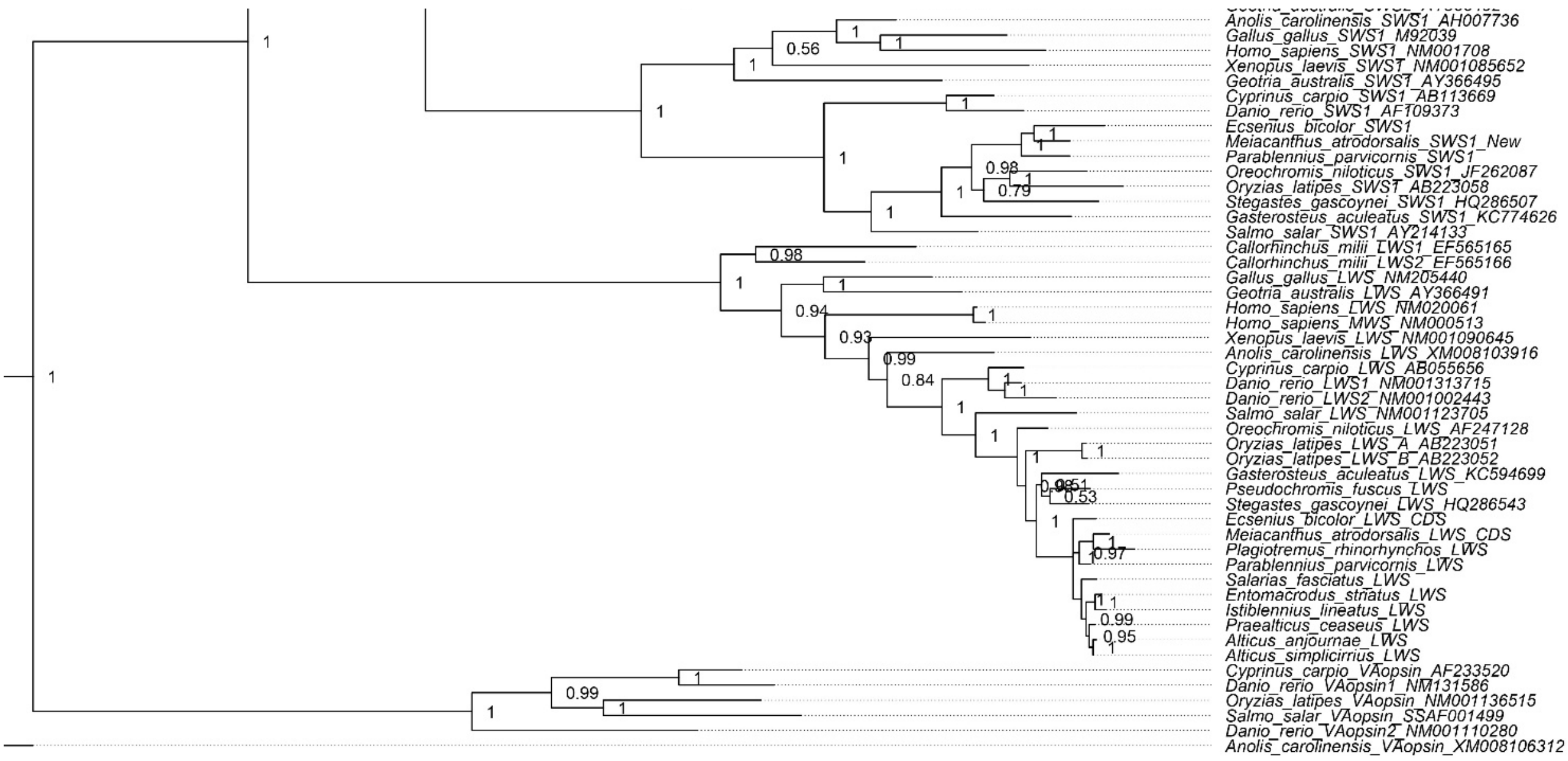

Biology Open • Supplementary information

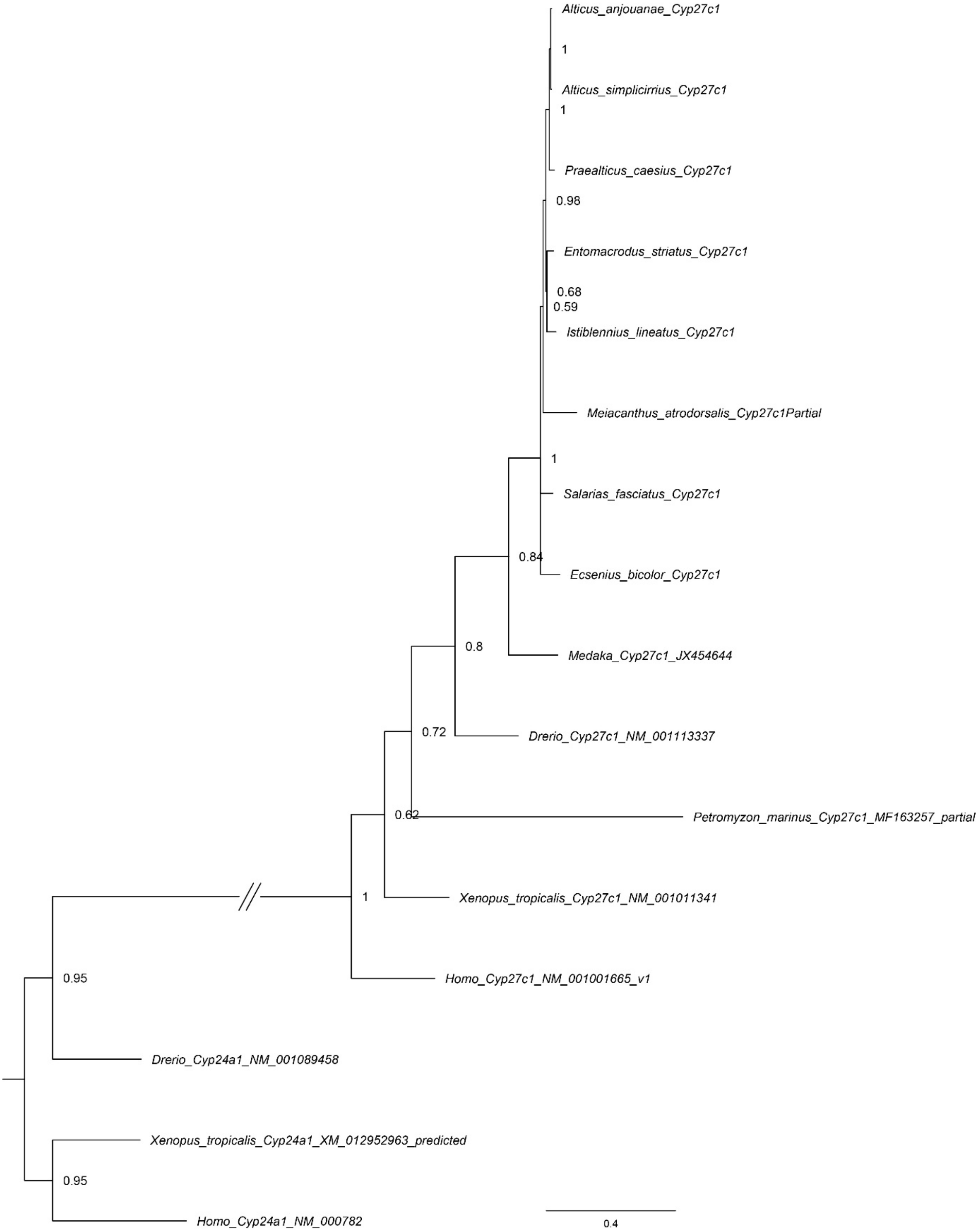

Fig. S2. Bayesian consensus phylogeny of the vertebrate *cypt27c1* gene. *Cyp27c1* has been shown to encode the enzyme that converts A_1 (rhodopsin) to the A_2 (porphyropsin) chromophore (Enright et al., 2015). Bayesian posterior probabilities are inset in bold.

Biology Open • Supplementary information

Supplementary References

Enright, J. M., M. B. Toomey, S.-y. Sato, S. E. Temple, J. R. Allen, R. Fujiwara, V. M. Kramlinger, L. D. Nagy, K. M. Johnson, Y. Xiao, M. J. How, S. L. Johnson, N. W. Roberts, V. J. Kefalov, P. F. Guengerich, and J. C. Corbo. 2015. Cyp27c1 red-shifts the spectral sensitivity of photoreceptors by converting vitamin A1 into A2. Current Biology **25**:3048-3057.

Musilova, Z., F. Cortesi, M. Matschiner, W. I. L. Davies, J. S. Patel, S. M. Stieb, F. de Busserolles, M. Malmstrøm, O. K. Tørresen, C. J. Brown, J. K. Mountford, R. Hanel, D. L. Stenkamp, K. S. Jakobsen, K. L. Carleton, S. Jentoft, J. Marshall, and W. Salzburger. 2019. Vision using multiple distinct rod opsins in deep-sea fishes. Science **364**: 588-592.

Stieb, S.M., F. Cortesi, L. Jardim De Queiroz, K. L. Carleton, O. Seehausen, and N. J. Marshall. 2023. Long-wavelength-sensitive (*lws*) opsin gene expression, foraging and visual communication in coral reef fishes. Molecular Ecology **32**:1656-1672.

Yang, Z. 2007. PAML 4: phylogenetic analysis by maximum likelihood. Molecular Biology and Evolution **24**:1586-1591.
