# Supplementary material for: Opsin gene evolution in aquatic, amphibious and terrestrial blennies (Blenniidae)
Source: Biol Open. 2026 Jul 15;15(7):bio062593. doi: 10.1242/bio.062593 (PMC13446567; doi:10.1242/bio.062593)
Supplement: Supplementary file 2 [file biolopen-15-062593_review_history.pdf]

Opsin gene evolution in aquatic, amphibious and terrestrial blennies (Blenniidae)

Karen L. Cheney, Georgina M. Schlub, Zuzana Musilova, Terry J. Ord and Fabio Cortesi
10.1242/bio.062593

Editor: Valentina di Santo

Review timeline

Original submission:	29 March 2026
Editorial decision:	7 April 2026
First revision received:	11 June 2026
Accepted:	15 June 2026

Original submission

First decision letter

MS ID#: bio.062593

MS Title: Opsin gene evolution in aquatic, amphibious and terrestrial blennies (Blenniidae)

Authors: Fabio Cortesi; Karen L. Cheney; Georgina M. Schlub; Zuzana Musilova; Terry J. Ord

I have now reached a decision on the above manuscript.

The reviewer reports are shown at the bottom of this email.

As you will see, the reviewers gave favourable reports, but raised some critical points that will require amendments to your manuscript. I hope that you will be able to carry these out, because we would like to be able to accept your paper.

At this stage, we also ask you to ensure your manuscript complies with our formatting guidelines - please see our manuscript preparation guidelines for details. Provided you are able to fully address the referees' comments, we are positive about publication of your paper (we accept over 95% of revision submissions) and therefore hope you won't mind any extra work involved in reformatting your manuscript at this point.

Please upload both a 'clean' version of your Word file, along with a highlighted version clearly showing where you have made changes in the revised manuscript. Please avoid using 'Track changes' in Word files as these are lost in PDF conversion.

I should be grateful if you would also provide a point-by-point response detailing how you have dealt with the points raised by the reviewers in the 'Response to Reviewers' box. Please attend to all of the reviewers' comments. If you do not agree with any of their criticisms or suggestions please explain clearly why this is so.

Reviewer 1

Comments for the author

© 2026. Published by The Company of Biologists under the terms of the Creative Commons Attribution License (https://creativecommons.org/licenses/by/4.0/).

In this manuscript, Cortesi et al. ask whether opsin genes are differentially regulated in the retina of different blennies species. The hypothesis is that depending on the habitat of the species, opsin genes expression and or sequences may have been selected to adapt to life on land or water.

To test this hypothesis, the authors selected 9 blennies. They obtained retinal transcriptomic data from the different species living on diverse habitats. Their main conclusions are that there is minimal sequence variation in opsin genes and no correlation between habitat and expression. They found trace expression of sws1 and sws2b, and showed that there is a strong phylogenetic inertia for opsin gene expression. Therefore, there is no evidence of habitat-driven opsin adaptation. Overall, the data is interesting depsite being mostly negative results and the analyses are well conducted.

The strength lies in the dataset that covers 9 species. The analyses are appropriate (PGLS, PAML). The weaknesses are that it is still a limited sample size, particularly because the sampling time is not uniform for all specimen. There is also no functional validation of the transcriptomic analyses. However, the authors identify these limits and discuss them in the text.

A minor comment also relates to the trace expression of sws1 and sws2b: one question that arise is whether these traces are due to individuals variations or if it is a consistent trace amount in all individuals?

Finally, the introduction could be shortened (for instance by removing the Anableps anableps example, or the morphological adaptations in Salarinii since the text is already focusing on opsin genes).

Reviewer 2

Comments for the author

In this study, Cortesi and colleagues investigate the expression of retinal opsin genes in fish of the Blennidae family and attempt to link gene expression to environmental adaptations by analyzing the retinal transcriptome of 9 different blenny species including fully aquatic, amphibious and predominantly terrestrial species. The analysis was focused on opsin genes, which code for photoreceptor proteins, crucial for vision.

The authors clearly state their hypothesis, which is that expression of opsin coding genes or amino-acid sequence of opsin proteins is driven by the amount of light to which the animals are exposed to, which crucially depends on their habitat. According to this logic, terrestrial species should have reduced expression of opsin genes linked to short wavelength spectral sensitivity (sws), resulting in a long wavelength-shifted photoreceptor sensitivity, compared to closely related amphibious species. This would be consistent with an adaptation to brighter light, which is richer in shorter spectral wavelengths (blue-UV) compared to the dimmer light of aquatic environments. These short wavelengths, particularly in the UV range, cause damage to the eyes and a reduced expression of sws genes could have evolved as a protective mechanism to filter out UV light.

For this study, the authors collected retinal tissue from 6 different blenny species:

Istiblennius lineatus (N=3 from French Polynesia, N=3 from Seychelles) -mostly aquatic

Praealticus caesius (N=3 from French Polynesia) - mildly amphibious

Alticus simplicirrus (N=3 from French Polynesia) - Terrestrial

Entomacrodus striatus (N=3 from Seychelles) - amphibious

Alticus anjouanae (N=3 from Seychelles) - Terrestrial

Salarias fasciatus (N=1 from Heron Island, Australia) - aquatic

© 2026. Published by The Company of Biologists under the terms of the Creative Commons Attribution License (https://creativecommons.org/licenses/by/4.0/).

Additionally, they used data from 3 more species that were previously collected for a different study:

Ecsenius bicolor (N=3 previously collected from Lizard Island, Australia) - aquatic

Meiacanthus atrodorsalis (N=3 previously collected from Lizard Island, Australia) - aquatic

Plagiotremus rhinorhynchus (N=3 previously collected from Lizard Island, Australia) - aquatic

From this retinal tissue, they performed bulk RNA sequencing and found that, in contrast to the initial hypothesis, opsin expression does not correlate with the degree of terrestrial lifestyle, but rather with phylogenetic distance. The authors then performed amino acid sequence prediction in the same opsin targets and found some specie-specific amino acid substitutions that lead to minor spectral shifts in sensitivity but did not find any amino acid substitutions under positive selection.

The study is original and explores an interesting question with relevance to evolutionary biology of the visual system, and the choice of addressing this question in several Blennidae species, which offer a variety of environmental adaptations, was clever. The methods and analyses used are appropriate for across-species comparison of gene expression. The results support the authors' conclusions and confirm a null hypothesis. Overall, the authors made great efforts to collect a variety of samples of different species, and to extract meaningful information, in line with the field standards. They provided sufficient context and cited relevant literature in the introduction. I appreciated the critical interpretation of results in the discussion, where the authors did a good job in framing the results in a broader context by discussing parallels with other fish families, and acknowledged the limitations of their study.

I only have minor comments.

* Data visualization can be improved. Opsin gene expression data was represented in a table. Given that opsin genes were the main focus of the study, I was expecting a more graphical representation, which is present in fig 2, but only in the form of a phylogenetic tree. Is there a way the author can represent the proportion of each opsin gene compared to its reference (es cone opsin vs total cone expression), for example using stacked bars? These are useful even with an N of 1-3 and I think this will convey the information in a more straightforward way.

* Quantification. The authors chose to quantify opsin genes in each sample comparing to total opsin expression within that sample. I wonder if there is another way of normalizing, using a reference that is common for all the species analyzed. I understand that the species used in the study have different genomes and perhaps do not have a common housekeeping gene that has consistent expression levels across the 9 species. Could this be done using the BUSCO genes mentioned in the methods for phylogenetic analyses? If one or more of these genes are conserved single copy genes with stable expression in retina across species, can opsin expression be represented as fold change against these genes as a reference? Perhaps it would not change the conclusions, but it would be a more robust normalization, accounting also for overall RNA integrity. If this is not possible, I think the choice of normalizing only against total opsin expression should be better motivated in the results section.

Reviewer's Responses to Questions

Experimental quality

Does each figure have the proper controls?
If 'No', please indicate reasons in Comments for Author box below.
Reviewer #1:

- Yes

Reviewer #2:

- Yes

© 2026. Published by The Company of Biologists under the terms of the Creative Commons Attribution License (https://creativecommons.org/licenses/by/4.0/).

Were the data analyzed using appropriate statistical tests?
If 'No', please indicate reasons in Comments for Author box below.
Reviewer #1:
- Yes

Reviewer #2:
- Yes

Reproducibility

Were experiments performed using adequate number of biological replicates?
If 'No', please indicate reasons in Comments for Author box below.
Reviewer #1:
- Yes

Reviewer #2:
- Yes

Does the methods section provide sufficient detail to permit reproducibility?
If 'No', please indicate reasons in Comments for Author box below.
Reviewer #1:
- Yes

Reviewer #2:
- Yes

Completeness

Are the manuscript's conclusions supported by the data?
If 'No', please indicate reasons in Comments for Author box below.
Reviewer #1:
- Yes

Reviewer #2:
- Yes

Scholarship

Do the authors cite and discuss the merits of data that would argue for and against their conclusion?
If 'No', please indicate reasons in Comments for Author box below.
Reviewer #1:
- Yes

Reviewer #2:
- Yes

Does the manuscript title & abstract accurately reflect the contents of the manuscript, without hyperbole?
If 'No', please indicate reasons in Comments for Author box below.
Reviewer #1:
- Yes

© 2026. Published by The Company of Biologists under the terms of the Creative Commons Attribution License (https://creativecommons.org/licenses/by/4.0/).

Reviewer #2:

- Yes

First revision

<u>Author response to reviewers' comments</u>

We thank both reviewers and the editor for their positive feedback and insights on our work. We have now gone through the comments and implemented the suggested amendments where possible. The manuscript has also been formatted according to the Biology Open instructions, with two versions: one highlighting the changes and a second, polished one attached.

We hope that our revisions are to the satisfaction of the journal and the reviewers, and we look forward to the manuscript being published shortly.

In addition to the Reviewer's comments, we have made several other changes:

- The affiliation for Dr Georgina Cooke/Schlub has been updated.
- The transcriptome (BioSample: SAMN60550786 – SAMN60550804) and single gene (PZ509097 – PZ509144) accession numbers have been added.
- The DOI for the supplementary data published on the UQ Research Data Manager has been added https://doi.org/10.48610/09af62a.
- We have added information about the taxonomists describing the species used in the study at first mention in the Methods section.

Reviewer 1: In this manuscript, Cortesi et al. ask whether opsin genes are differentially regulated in the retina of different blennies species. The hypothesis is that depending on the habitat of the species, opsin genes expression and or sequences may have been selected to adapt to life on land or water.

To test this hypothesis, the authors selected 9 blennies. They obtained retinal transcriptomic data from the different species living on diverse habitats. Their main conclusions are that there is minimal sequence variation in opsin genes and no correlation between habitat and expression. They found trace expression of *sws1* and *sws2b*, and showed that there is a strong phylogenetic inertia for opsin gene expression. Therefore, there is no evidence of habitat-driven opsin adaptation. Overall, the data is interesting depsite being mostly negative results and the analyses are well conducted.

The strength lies in the dataset that covers 9 species. The analyses are appropriate (PGLS, PAML). The weaknesses are that it is still a limited sample size, particularly because the sampling time is not uniform for all specimen. There is also no functional validation of the transcriptomic analyses. However, the authors identify these limits and discuss them in the text.
A minor comment also relates to the trace expression of *sws1* and *sws2b*: one question that arise is whether these traces are due to individuals variations or if it is a consistent trace amount in all individuals?

> The trace expression is a phenomenon we observe across fish species, with slight individual variation. These low levels of *sws1* opsin gene expression [SWS1 = UV-sensitive; note that there was no *sws2b* expression as this gene has likely been lost in the blenny ancestor (Cortesi et al., 2015)] are likely a remnant, or rather residual expression, from the larval stage, when the fish are exposed to high-UV environments and feeding on UV-reflecting or absorbing zooplankton. As the retina in a fish's eye continuously grows throughout its life, new photoreceptor cells are added by growing along the falciform process and then being added at the perimeter. Hence, the larval setup remains at the centre of the retina, represented by a small number of cells that often express a different opsin complement than that used in the adults, giving rise to the trace amounts of 'other' opsin expression. To the best of our knowledge, no study has yet assessed whether the larval cells remain functional in adults.

© 2026. Published by The Company of Biologists under the terms of the Creative Commons Attribution License (https://creativecommons.org/licenses/by/4.0/).

> We have added a sentence (L363 – 365) to explain the likely origin of the trace *sws1* opsin gene expression in the main text.

Finally, the introduction could be shortened (for instance, by removing the *Anableps anableps* example, or the morphological adaptations in Salarinii, since the text is already focusing on opsin genes).

> We respectfully disagree with the reviewer on this point. The sections on morphological adaptations of the visual system in amphibious fishes, including the four-eyed fish (*Anableps anableps*) (L64 – 74) and some Salariini blennies (L129 – 139), inform the reader that, when transitioning from water to land, fish vision is likely to change. Therefore, while morphological modifications have been documented, molecular adaptations are also likely, which is what we set out to investigate.

Reviewer 2: In this study, Cortesi and colleagues investigate the expression of retinal opsin genes in fish of the Blennidae family and attempt to link gene expression to environmental adaptations by analyzing the retinal transcriptome of 9 different blenny species including fully aquatic, amphibious and predominantly terrestrial species. The analysis was focused on opsin genes, which code for photoreceptor proteins, crucial for vision.

The authors clearly state their hypothesis, which is that expression of opsin coding genes or amino-acid sequence of opsin proteins is driven by the amount of light to which the animals are exposed to, which crucially depends on their habitat. According to this logic, terrestrial species should have reduced expression of opsin genes linked to short wavelength spectral sensitivity (sws), resulting in a long wavelength-shifted photoreceptor sensitivity, compared to closely related amphibious species. This would be consistent with an adaptation to brighter light, which is richer in shorter spectral wavelengths (blue-UV) compared to the dimmer light of aquatic environments. These short wavelengths, particularly in the UV range, cause damage to the eyes and a reduced expression of sws genes could have evolved as a protective mechanism to filter out UV light.

For this study, the authors collected retinal tissue from 6 different blenny species:
Istiblennius lineatus (N=3 from French Polynesia, N=3 from Seychelles) -mostly aquatic
Praealticus caesius (N=3 from French Polynesia) - mildly amphibious
Alticus simplicirrus (N=3 from French Polynesia) – Terrestrial
Entomacrodus striatus (N=3 from Seychelles) – amphibious
Alticus anjouanae (N=3 from Seychelles) – Terrestrial
Salarias fasciatus (N=1 from Heron Island, Australia) – aquatic

Additionally, they used data from 3 more species that were previously collected for a different study:
Ecsenius bicolor (N=3 previously collected from Lizard Island, Australia) – aquatic
Meiacanthus atrodorsalis (N=3 previously collected from Lizard Island, Australia) – aquatic
Plagiotremus rhinorhynchus (N=3 previously collected from Lizard Island, Australia) – aquatic

From this retinal tissue, they performed bulk RNA sequencing and found that, in contrast to the initial hypothesis, opsin expression does not correlate with the degree of terrestrial lifestyle, but rather with phylogenetic distance. The authors then performed amino acid sequence prediction in the same opsin targets and found some specie-specific amino acid substitutions that lead to minor spectral shifts in sensitivity but did not find any amino acid substitutions under positive selection.

The study is original and explores an interesting question with relevance to evolutionary biology of the visual system, and the choice of addressing this question in several Blennidae species, which offer a variety of environmental adaptations, was clever. The methods and analyses used are appropriate for across-species comparison of gene expression. The results support the authors' conclusions and confirm a null hypothesis. Overall, the authors made great efforts to collect a variety of samples of different species, and to extract meaningful information, in line with the field standards. They provided sufficient context and cited relevant literature in the introduction. I appreciated the critical interpretation of results in the discussion, where the authors did a good job

© 2026. Published by The Company of Biologists under the terms of the Creative Commons Attribution License (https://creativecommons.org/licenses/by/4.0/).

in framing the results in a broader context by discussing parallels with other fish families, and acknowledged the limitations of their study.

I only have minor comments.

* Data visualization can be improved. Opsin gene expression data was represented in a table. Given that opsin genes were the main focus of the study, I was expecting a more graphical representation, which is present in fig 2, but only in the form of a phylogenetic tree. Is there a way the author can represent the proportion of each opsin gene compared to its reference (es cone opsin vs total cone expression), for example using stacked bars? These are useful even with an N of 1-3 and I think this will convey the information in a more straightforward way.

> We have modified Figure 1 as suggested, adding stacked bars to the species phylogeny to show the proportional expression of single and double cones. The figure legend has been changed accordingly.

* Quantification. The authors chose to quantify opsin genes in each sample comparing to total opsin expression within that sample. I wonder if there is another way of normalizing, using a reference that is common for all the species analyzed. I understand that the species used in the study have different genomes and perhaps do not have a common housekeeping gene that has consistent expression levels across the 9 species. Could this be done using the BUSCO genes mentioned in the methods for phylogenetic analyses? If one or more of these genes are conserved single copy genes with stable expression in retina across species, can opsin expression be represented as fold change against these genes as a reference? Perhaps it would not change the conclusions, but it would be a more robust normalization, accounting also for overall RNA integrity. If this is not possible, I think the choice of normalizing only against total opsin expression should be better motivated in the results section.

> This is an excellent observation. The conventional approach to quantify gene expression is to normalise it to the expression of several housekeeping genes, to the transcriptome size (FPKM) or to the specific gene expression of interest across the transcriptome (TPM). However, for genes that show strong circadian rhythmicity, such as the visual opsin genes, this relative expression can only be calculated if sample collection is standardised to the same time of day (ideally, early afternoon for cone opsin expression). Unfortunately, this was not the case in our study.

Because cone opsin expression is synchronised across the day, calculating each gene's expression as a proportion of total cone opsin expression is an alternative. While it does not provide information about changes in the absolute numbers of reads between species or over time, it does convey changes in colour vision, i.e., whether an individual uses more of a specific opsin than another, which was what we were interested in. Yourick and colleagues (2019) published a seminal article on the topic, measuring opsin gene expression in cichlid fishes over a 24 h period. We have added this information to the Methods section (L268 –276) to explain our reasoning more clearly.

Second decision letter

MS ID#: bio.062593R1

MS Title: Opsin gene evolution in aquatic, amphibious and terrestrial blennies (Blenniidae)

Authors: Fabio Cortesi; Karen L. Cheney; Georgina M. Schlub; Zuzana Musilova; Terry J. Ord

I am happy to tell you that your manuscript has been accepted for publication in Biology Open, pending our standard publication integrity checks. It was accepted on 15th June 2026.

© 2026. Published by The Company of Biologists under the terms of the Creative Commons Attribution License (https://creativecommons.org/licenses/by/4.0/).
